# Acute Heart Failure and Non-Ischemic Cardiomyopathies: A Comprehensive Review and Critical Appraisal

**DOI:** 10.3390/diagnostics15050540

**Published:** 2025-02-23

**Authors:** Lina Manzi, Federica Buongiorno, Viviana Narciso, Domenico Florimonte, Imma Forzano, Domenico Simone Castiello, Luca Sperandeo, Roberta Paolillo, Nicola Verde, Alessandra Spinelli, Stefano Cristiano, Marisa Avvedimento, Mario Enrico Canonico, Luca Bardi, Giuseppe Giugliano, Giuseppe Gargiulo

**Affiliations:** 1 Department of Advanced Biomedical Sciences, Federico II University of Naples, 80131 Naples, Italy; lina.manzi93@gmail.com (L.M.); federicabuongiorno2@gmail.com (F.B.); viviana.narciso@gmail.com (V.N.); florimontedomenico@gmail.com (D.F.); imma.forzano@gmail.com (I.F.); ds.castiello@gmail.com (D.S.C.); luca.sperandeo95@gmail.com (L.S.); robe.paolillo@gmail.com (R.P.); nicoverde91@gmail.com (N.V.); alessandra.spinelli@unina.it (A.S.); stefano.cristiano@unina.it (S.C.); m.avvedimento@gmail.com (M.A.); marioenrico.canonico@unina.it (M.E.C.); bardiluca@me.com (L.B.); giuseppe.giugliano@unina.it (G.G.); 2 Department of Cardiology, AORN Cardarelli, 80131 Naples, Italy

**Keywords:** acute heart failure, cardiomyopathies, dilated cardiomyopathy, hypertrophic cardiomyopathy, restrictive cardiomyopathy, non-dilated left ventricular cardiomyopathy, arrhythmogenic right ventricular cardiomyopathy

## Abstract

Acute heart failure (AHF) is a complex clinical syndrome characterized by the rapid or gradual onset of symptoms and/or signs of heart failure (HF), leading to an unplanned hospital admission or an emergency department visit. AHF is the leading cause of hospitalization in patients over 65 years, thus significantly impacting public health care. However, its prognosis remains poor with high rates of mortality and rehospitalization. Many pre-existing cardiac conditions can lead to AHF, but it can also arise de novo due to acute events. Therefore, understanding AHF etiology could improve patient management and outcomes. Cardiomyopathies (CMPs) are a heterogeneous group of heart muscle diseases, including dilated cardiomyopathy (DCM), hypertrophic cardiomyopathy (HCM), restrictive cardiomyopathy (RCM), non-dilated cardiomyopathy (NDLVC), and arrhythmogenic right ventricular cardiomyopathy (ARVC), that frequently present with HF. Patients with CMPs are under-represented in AHF studies compared to other etiologies, and therefore therapeutic responses and prognoses remain unknown. In DCM, AHF represents the most frequent cause of death despite treatment improvements. Additionally, DCM is the first indication for heart transplant (HT) among young and middle-aged adults. In HCM, the progression to AHF is rare and more frequent in patients with concomitant severe left ventricle (LV) obstruction and hypertrophy or severe LV systolic dysfunction. HF is the natural evolution of patients with RCM and HF is associated with poor outcomes irrespective of RCM etiology. Furthermore, while the occurrence of AHF is rare among patients with ARVC, this condition in NDLVC patients is currently unknown. In this manuscript, we assessed the available evidence on AHF in patients with CMPs. Data on clinical presentation, therapeutic management, and clinical outcomes according to specific CMPs are limited. Future HF studies assessing the clinical presentation, treatment, and prognosis of specific CMPs are warranted.

## 1. Introduction

**Figure 1:**
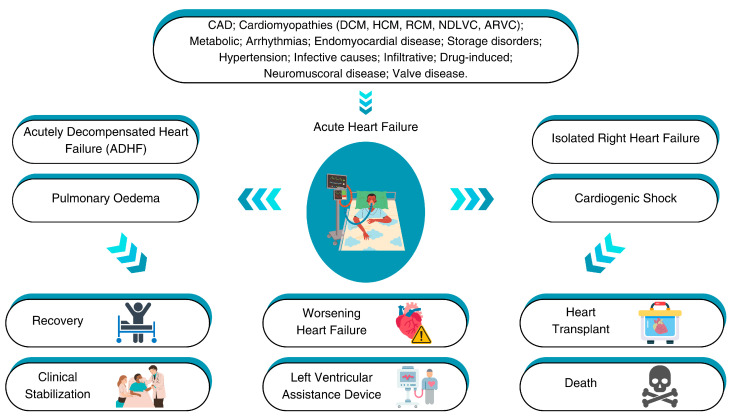
Etiology, clinical presentation, and outcomes of acute heart failure. The figure summarizes the multiple clinical conditions that may determine AHF, as well as the various clinical presentations of a patient with AHF, and this variability is inevitably associated with different prognostic implications. Abbreviations: ARVC = arrhythmogenic right ventricular cardiomyopathy; CAD = coronary artery disease; DCM = dilated cardiomyopathy; HCM = hypertrophic cardiomyopathy; NDLVC = non-dilated left ventricular cardiomyopathy; RCM = restrictive cardiomyopathy.

Acute heart failure (AHF) is a complex syndrome characterized by the rapid onset or worsening of symptoms and signs of heart failure (HF), necessitating urgent medical intervention. AHF is the leading cause of unplanned hospital admissions among individuals over 65 years. However, the prognosis remains poor, with high rates of mortality and rehospitalization. Patients with pre-existing cardiac diseases could develop AHF, but it can also occur de novo in response to acute events. Given its prevalence and prognosis, understanding AHF etiology is essential to improving patient outcomes and reducing associated healthcare costs (Figure 1) [1,2]. Furthermore, patients with cardiomyopathies (CMPs), a heterogeneous group of heart muscle diseases, including dilated cardiomyopathy (DCM), hypertrophic cardiomyopathy (HCM), restrictive cardiomyopathy (RCM), non-dilated cardiomyopathy (NDLVC), and arrhythmogenic right ventricular cardiomyopathy (ARVC), may develop AHF. However, when compared to other etiologies of HF, the therapeutic response and prognosis of patients affected by CMPs remain unknown. The aim of this article is to review the evidence on AHF in patients with non-ischemic CMPs and to summarize the data from the main observational and randomized trials on AHF, highlighting the differences according to patient characteristics.

## 2. Acute Heart Failure

AHF refers to the rapid or gradual onset of symptoms and/or signs of HF severe enough for the patient to seek urgent medical attention, leading to an unplanned hospital admission or an emergency department visit. Patients with AHF require urgent evaluation with subsequent initiation and/or intensification of treatment, including IV therapies or procedures [1].

### 2.1. Clinical Presentation

Four clinical presentations of AHF can be described, with possible overlap among them. According to the European Society of Cardiology (ESC) Heart Failure Long-Term Registry, 61% of AHF patients present with acutely decompensated heart failure (ADHF), 13% with acute pulmonary edema, 3.5% with isolated right ventricular (RV) failure, and 3% with cardiogenic shock (CS) [3].

Generally, patients with ADHF have a worsening of chronic HF and have moderately to severely reduced left ventricular (LV) systolic function [4]. These patients often present with systemic congestion with or without hypotension [5]. Acute pulmonary edema is due to fluid redistribution to the lungs, leading to acute respiratory failure. It has a faster onset compared to ADHF and is a life-threatening condition if not promptly treated with oxygen support, diuretics, vasodilators, or inotropes/vasopressors [6]. Isolated RV failure is characterized by increased RV and atrial pressure, RV dysfunction, and systemic congestion, finally leading to a reduction in LV cardiac output (CO) through ventricular interdependence. RV failure is mainly caused by a pulmonary embolism (PE), right myocardial infarction (MI), or myocarditis [7]. Finally, CS is the worst clinical presentation, characterized by inadequate CO, leading to a life-threatening state of tissue hypoperfusion that can evolve into multi-organ failure and death [8,9,10].

### 2.2. Epidemiology and Prognosis

AHF represents the most common cause of hospitalization in individuals aged 65 years or older in Western countries, accounting for >1 million hospitalizations per year in the United States. AHF causes substantial morbidity and mortality and is a remarkable burden on health economics worldwide [11]. The prevalence of AHF is approximately 1–2% and increases to >10% among older people (>70 years); however, these estimates may vary according to the study population [12]. The prognosis is often poor and AHF is associated with high mortality and rehospitalization rates. In-hospital mortality ranges from 4% to 10% and death or rehospitalization rates may reach 45% [3,5,13]. This poor prognosis is also related to the characteristics of the people admitted for HF, who are often old, with relevant comorbidities and high rates of admissions due to non-cardiac reasons. Therefore, strengthening continuity of care and managing comorbidities to prevent acute conditions are needed.

### 2.3. Etiology

Most AHF patients (65–75%) have a known history of HF or cardiovascular disease (CVD). Around 50% of patients are male and have arterial hypertension (70%), coronary artery disease (CAD) (50–60%), or atrial fibrillation (AF) (30–40%). Non-cardiovascular comorbidities include diabetes mellitus in approximately 40% of patients, renal dysfunction in 20–30%, chronic obstructive pulmonary disease (COPD) in 20–30%, and anemia in 15–30% [1]. A systematic review of worldwide risk factors for HF showed that CAD is the major underlying contributor to AHF admissions in >50% of patients in high-income regions, as well as eastern and central European regions. In Asia and Latin America, CAD contributes to 30–40% of admissions, while it contributes to <10% in sub-Saharan Africa [14]. For a practical and rapid assessment, when a patient with AHF is admitted to hospital, specific causes can be addressed with the CHAMPIT acronym (acute coronary syndrome (ACS), hypertensive emergency, arrhythmias, acute mechanical cause, PE, infection, and tamponade) [1]. Once these specific etiologies are excluded, the management of AHF should be individualized according to clinical presentation and phenotype.

### 2.4. Evidence on AHF in Observational Studies

We searched for data regarding the incidence, prevalence, etiology, and outcome of AHF patients, trying to investigate the key differences in terms of outcomes in patients with a non-ischemic AHF etiology. The ADHERE (Acute Decompensated Heart Failure National Registry) registry enrolled 65,180 patients through 263 hospitals in the United States. The median age of patients was 75.2 years, and more than half (52%) were female. Most patients (75%) had a history of HF, 23% had been hospitalized for AHF within the prior 6 months, and 3% had three or more HF-related hospitalizations in that period. Regarding medical history, 72% had a history of arterial hypertension, while other common conditions were CAD (58%), diabetes (44%), AF (31%), and COPD or asthma (31%) [15]. The OPTIMIZE-HF (Organized Program to Initiate Lifesaving Treatment in Hospitalized Patients with Heart Failure) registry included 48,612 patients, of whom 52% were women and 74% were Caucasian. Their underlying diseases were CAD (in 46% of all patients), AF (in 32%), COPD (in 28%), arterial hypertension (in 23%), insulin-independent diabetes (in 25%), and insulin-dependent diabetes (in 17%). Interestingly, multivariable predictors of mortality included age, heart rate, systolic blood pressure (SBP), sodium, creatinine, HF as the primary cause of hospitalization, and the presence of LV systolic dysfunction [16]. The AHEAD (Acute Heart Failure Database) registry comprised patients hospitalized for AHF in seven Czech Republic hospitals. Of 4153 patients, the most frequent etiologies were ACS (36.2%), chronic coronary syndrome (CCS) (19.9%), valvular disease (10.4%), arrhythmias (7.9%), and arterial hypertension (5.7%). Patients with ACS had lower mortality than those without ACS (9.7% versus 18.1%), while patients with CS had the highest mortality (62.7%) [11]. The EHFS II (EuroHeart Failure Survey II) is a European survey that included 3580 patients hospitalized for AHF. The most common underlying diseases were arterial hypertension (62%), CAD (53.6%), AF (38%), valvular disease (34%), and diabetes mellitus (32%). Concomitant precipitant diseases were ACS (30%), arrhythmias (32%), valvular cause (26%), infection (17%), and non-compliance to therapy (22%) [13]. The international ALARM (Acute Heart Failure Global Registry of Standard Treatment) registry described the characteristics and management of AHF among various countries in order to compare patients with de novo AHF to patients with a pre-existing episode of AHF. The most common clinical presentation was ADHF (38.6%), followed by pulmonary edema (36.7%), CS (11.7%), hypertensive HF (7.4%), right HF (4.5%), and high-output HF (1.1%) The most common underlying diseases were hypertension (70%), diabetes mellitus (45%), AF (38%), chronic HF (37%), CAD (30%), COPD (24%), chronic renal disease (21%), and non-ischemic CMPs (12.6%), while concomitant precipitant diseases were ACS (37%), arrhythmias (27%), infection (16%), and non-compliance with therapy (13%) [17]. In a real-world cohort of 47,241 patients with heart failure with reduced ejection fraction (HFrEF) from the Swedish Heart Failure Registry (SwedeHF), hospitalization with first-episode HF and a higher risk of CV mortality were more frequent in patients with a concomitant glomerular filtration rate (eGFR) of 30–60 mL/min/1.73 m^2^ and AF. Furthermore, the authors identified nine profiles with the highest event rates, representing only 5% of the study population, characterized by no hyperkaliemia, an even distribution among the SBP strata, eGFR <30 mL/min/1.73 m^2^, and AF [18]. A recent multicenter retrospective study focused on the management of CS with the use of mechanical circulatory support (MCS) in non-ischemic patients. In these settings, the use of such devices was associated with lower 30-day mortality (HR 0.76, 95% CI 0.59–0.97); however, it was also associated with more complications (severe bleeding (16.5% for MCS vs. 6.4% for non-MCS patients) and access-site-related ischemia (6.7% for MCS vs. 0% for non-MCS patients). Surprisingly, although specific data on non-ischemic CS patients are of paramount importance, no data were reported on the characterization of these patients and details of their etiologies [19].

What emerges from these registries is the fact that patients with AHF are very heterogeneous and this reflects the differences in prognosis. CAD is the most common primary cause in patients with AHF, while ACS is a frequent precipitant factor. In the context of de novo HF, a large amount of evidence suggests that it may have a worse prognosis compared to ADHF. Indeed, in ALARM HF, in-hospital mortality was 12%, the majority of which was due to CS (43%) and in patients with de novo AHF (14.2%). In addition, in the EHFS II registry (overall in-hospital mortality of 6.7%), de novo AHF patients had higher in-hospital mortality (8.1%). In AHEAD, the overall hospital mortality of 12.7% was comparable with the mortality of ALARM-HF, while in-hospital mortality rates for ADHERE (4%) and OPTIMIZE-HF (3.8%) were similar [11,13,15,17].

### 2.5. Evidence on AHF in Randomized Clinical Trials

Studying AHF as a unique entity is challenging and represents a problem when trying to obtain data from trials that should fit all AHF patients. We attempted to summarize the data from the main AHF trials, highlighting the differences in patient characteristics. Table 1 reports details of the main randomized AHF trials. Here, we mainly focused on the data on patients with non-ischemic CMPs. In the ADVOR (acetazolamide in decompensated heart failure with volume overload) trial, the underlying diseases of patients reflected the ones of the registries discussed before (43% CAD, 20% valvular heart disease, 24% DCM, and 9% arterial hypertension) [20]. In the CLOROTIC (combining loop with thiazide diuretics for decompensated heart failure) trial, the patient population had a high burden of comorbidities and high-risk features: diabetes (56.9% and 56.1% in HCTZ and placebo, respectively), AF (62.9% and 74.6%, respectively), anemia (45.7% and 43.9%, respectively), CAD (25.2% and 40.4%, respectively), and COPD (21.6% and 23.7%, respectively). Interestingly, in the subgroup analysis, patients with both ischemic and non-ischemic CMPs benefitted from the addition of hydrochlorothiazide, although non-ischemic patients showed a better response [21]. Most patients in the COACH (in the comparison of outcomes and access to care for heart failure) trial had a history of HF (63.5%) or hypertension (75.2%); however, it is not known whether non-ischemic HF patients had a better prognosis and earlier discharge [22]. In the EMPULSE (EMPagliflozin outcome trial in patients hospitalized for acute heart failure with preserved and reduced ejection fraction) trial, although clinical benefits were observed for both AHF and ADHF irrespective of LV ejection fraction (LVEF), there are no data about the role of gliflozins in AHF patients based on etiology [23]. In the AFFIRM-AHF (a randomized, double-blind placebo-controlled trial comparing the effect of intravenous ferric carboxymaltose on hospitalizations and mortality in iron-deficient subjects admitted for acute heart failure) trial, 71.3% of patients had a previous history of HF and 47.1% had an ischemic CMP. Furthermore, in the subgroup analysis, ischemic HF patients benefitted more from iron administration compared to non-ischemic HF patients. Therefore, HF etiology could be an effect modifier, given that non-ischemic HF etiology comprises a heterogeneous group of patients, in whom the pathophysiological consequences of iron deficiency are not yet well-established [24]. In addition, the ASTRONAUT (the aliskiren trial on acute heart failure outcomes) trial included predominantly ischemic patients (63.6%) rather than non-ischemic patients. Furthermore, in the prespecified subgroup analysis of the primary endpoint, the negative finding was consistent across ischemic and non-ischemic groups [25]. In the STRONG-HF (the safety, tolerability, and efficacy of rapid optimization, helped by NT-proBNP testing, of heart failure therapies) trial, the percentages of patients with ischemic vs. non-ischemic CMPs were similar (48% and 52%, respectively); however, no data on outcomes according to etiology are available [26]. In the PIONEER (comparison of sacubitril–valsartan versus enalapril on effect on NT-proBNP in patients stabilized from an acute heart failure episode) trial, the baseline characteristics of the enrolled patients were: previous MI (7%), hyperlipidemia (37.1%), hypertension (85.5%), diabetes mellitus (19.1%), previous stroke (9.9%), AF (35.4%), chronic renal insufficiency (28.3%), and current smoking (22.5%). However, there is no clear etiology reported and no data on the effectiveness of sacubitril–valsartan according to HF etiologies in patients with AHF [27]. The subgroup analysis of SURVIVE (survival of patients with acute heart failure in need of intravenous inotropic support) trial revealed that a prior history of HF at baseline influenced the between-group difference at 31 days (treatment x prior HF interaction, *p* = 0.05) but not at 180 days, while MI as a primary cause of hospitalization did not influence the between-group difference at 31 days and 180 days (treatment x prior HF interaction, *p* = 0.82 and *p* = 0.23, respectively) [28]. In the CHAMPION (CardioMEMS heart sensor allows monitoring of pressure to improve outcomes in NYHA class III heart failure patients) trial, the comorbidities of the population were hypertension (77% vs. 79%, respectively in the treatment vs. placebo groups), CAD (67% vs. 72%), diabetes mellitus (48% vs. 50%), atrial flutter or fibrillation (44% vs. 48%), COPD (28% vs. 30%), and chronic kidney disease (20% vs. 19%), but again, the outcomes of the different etiologies of HF are unknown [29]. The subgroup analysis of CV death in the TRUE-AHF (ularitide efficacy and safety in acute heart failure) trial explored the drug’s effect according to the presence or absence of CAD history, with a negative interaction suggesting that the lack of a significant benefit of ularitide was consistent regardless of AHF etiology [30]. The RELAX-AHF 2 (effects of serelaxin in patients with acute heart failure) trial also reported the percentage of ischemic patients with AHF (54.5% vs. 53.1, respectively, in serelaxin vs. placebo groups), although outcomes stratified by etiology are not reported [31]. In a post hoc subgroup analysis of the SOLOIST-WHF (sotagliflozin in patients with diabetes and recent worsening heart failure) trial, the primary endpoint was assessed according to the etiology of HF, comparing the drug effect in patients with ischemic versus non-ischemic HF, suggesting that the greatest benefit was observed in ischemic HF (*n* = 712 vs. 503; HR (95% CI) for sotagliflozin vs. placebo 0.55; 0.41, 0.74 vs. 0.88; 0.59, 1.31) [32]. Surprisingly, in the REVIVE I and II (effect of levosimendan on the short-term clinical course of patients with acutely decompensated heart failure), GALACTIC (effect of a strategy of comprehensive vasodilation vs. usual care on mortality and heart failure rehospitalization among patients with acute heart failure), and VICTORIA (vericiguat in patients with heart failure and reduced ejection fraction) trials, there are no data on differences in outcomes between ischemic vs. non-ischemic HF patients [33,34,35]. On the contrary, the LeoDOR (repetitive levosimendan infusions for patients with advanced chronic heart failure in the weakening post-discharge period) trial reports the percentages of patients with non-ischemic HF treated with levosimendan compared with placebo (ischemic HF 38.7% vs. 50%, *p* = 0.127; DCM 69.9% vs. 53.8%, *p* = 0.04; restrictive cardiomyopathy (RCM) 2.2% vs. 0% *p* = 0.410) [36]. In the subgroup analyses of the PUSH-AHF (natriuresis-guided diuretic therapy in acute heart failure: a pragmatic randomized trial) trial, the main findings (better natriuresis without significant benefit in clinical endpoints) were consistent across subgroups based on ischemic or non-ischemic etiology with negative interaction *p*-values for both dual primary endpoints (24 h urinary sodium excretion and a combined endpoint of time to all-cause mortality or adjudicated heart failure rehospitalization at 180 days) [37]. In the subgroup analysis of the PREMIER (in-hospital initiation of angiotensin receptor–neprilysin inhibition in acute heart failure) trial, the benefit of sacubitril/valsartan on NT-proBNP levels at week 8 was consistent in patients with ischemic and non-ischemic HF (*p* for interaction = 0.781) [38]. In a subgroup analysis of the UNLOAD (ultrafiltration versus intravenous diuretics for patients hospitalized for acute decompensated heart failure) trial, compared with intravenous diuretics, ultrafiltration resulted in lower HF rehospitalization rates, and this benefit seemed to be greater in non-ischemic HF patients than in ischemic HF patients (HR 0.21, CI: 0.05–1.00 vs. 0.72, CI: 0.33–1.56) [39]. In the CUORE (continuous ultrafiltration for congestive heart failure) trial, only the percentages of ischemic HF vs. non-ischemic HF patients in the control vs. ultrafiltration groups were reported (55% vs. 59%, *p* = 0.55; 17% vs. 15%, *p* = 0.81) [40].

Studying AHF as a unique entity is challenging and represents a problem when trying to obtain data from trials that should fit all AHF patients. We attempted to summarize the data from the main AHF trials, highlighting the differences in patient characteristics. Table 1 reports details of the main randomized AHF trials. Here, we mainly focused on the data on patients with non-ischemic CMPs. In the ADVOR (acetazolamide in decompensated heart failure with volume overload) trial, the underlying diseases of patients reflected the ones of the registries discussed before (43% CAD, 20% valvular heart disease, 24% DCM, and 9% arterial hypertension) [20]. In the CLOROTIC (combining loop with thiazide diuretics for decompensated heart failure) trial, the patient population had a high burden of comorbidities and high-risk features: diabetes (56.9% and 56.1% in HCTZ and placebo, respectively), AF (62.9% and 74.6%, respectively), anemia (45.7% and 43.9%, respectively), CAD (25.2% and 40.4%, respectively), and COPD (21.6% and 23.7%, respectively). Interestingly, in the subgroup analysis, patients with both ischemic and non-ischemic CMPs benefitted from the addition of hydrochlorothiazide, although non-ischemic patients showed a better response [21]. Most patients in the COACH (in the comparison of outcomes and access to care for heart failure) trial had a history of HF (63.5%) or hypertension (75.2%); however, it is not known whether non-ischemic HF patients had a better prognosis and earlier discharge [22]. In the EMPULSE (EMPagliflozin outcome trial in patients hospitalized for acute heart failure with preserved and reduced ejection fraction) trial, although clinical benefits were observed for both AHF and ADHF irrespective of LV ejection fraction (LVEF), there are no data about the role of gliflozins in AHF patients based on etiology [23]. In the AFFIRM-AHF (a randomized, double-blind placebo-controlled trial comparing the effect of intravenous ferric carboxymaltose on hospitalizations and mortality in iron-deficient subjects admitted for acute heart failure) trial, 71.3% of patients had a previous history of HF and 47.1% had an ischemic CMP. Furthermore, in the subgroup analysis, ischemic HF patients benefitted more from iron administration compared to non-ischemic HF patients. Therefore, HF etiology could be an effect modifier, given that non-ischemic HF etiology comprises a heterogeneous group of patients, in whom the pathophysiological consequences of iron deficiency are not yet well-established [24]. In addition, the ASTRONAUT (the aliskiren trial on acute heart failure outcomes) trial included predominantly ischemic patients (63.6%) rather than non-ischemic patients. Furthermore, in the prespecified subgroup analysis of the primary endpoint, the negative finding was consistent across ischemic and non-ischemic groups [25]. In the STRONG-HF (the safety, tolerability, and efficacy of rapid optimization, helped by NT-proBNP testing, of heart failure therapies) trial, the percentages of patients with ischemic vs. non-ischemic CMPs were similar (48% and 52%, respectively); however, no data on outcomes according to etiology are available [26]. In the PIONEER (comparison of sacubitril–valsartan versus enalapril on effect on NT-proBNP in patients stabilized from an acute heart failure episode) trial, the baseline characteristics of the enrolled patients were: previous MI (7%), hyperlipidemia (37.1%), hypertension (85.5%), diabetes mellitus (19.1%), previous stroke (9.9%), AF (35.4%), chronic renal insufficiency (28.%), and current smoking (22.5%). However, there is no clear etiology reported and no data on the effectiveness of sacubitril–valsartan according to HF etiologies in patients with AHF [27]. The subgroup analysis of SURVIVE (survival of patients with acute heart failure in need of intravenous inotropic support) trial revealed that a prior history of HF at baseline influenced the between-group difference at 31 days (treatment x prior HF interaction, *p* = 0.05) but not at 180 days, while MI as a primary cause of hospitalization did not influence the between-group difference at 31 days and 180 days (treatment x prior HF interaction, *p* = 0.82 and *p* = 0.23, respectively) [28]. In the CHAMPION (CardioMEMS heart sensor allows monitoring of pressure to improve outcomes in NYHA class III heart failure patients) trial, the comorbidities of the population were hypertension (77% vs. 79%, respectively in the treatment vs. placebo groups), CAD (67% vs. 72%), diabetes mellitus (48% vs. 50%), atrial flutter or fibrillation (44% vs. 48%), COPD (28% vs. 30%), and chronic kidney disease (20% vs. 19%), but again, the outcomes of the different etiologies of HF are unknown [29]. The subgroup analysis of CV death in the TRUE-AHF (ularitide efficacy and safety in acute heart failure) trial explored the drug’s effect according to the presence or absence of CAD history, with a negative interaction suggesting that the lack of a significant benefit of ularitide was consistent regardless of AHF etiology [30]. The RELAX-AHF 2 (effects of serelaxin in patients with acute heart failure) trial also reported the percentage of ischemic patients with AHF (54.5% vs. 53.1, respectively, in serelaxin vs. placebo groups), although outcomes stratified by etiology are not reported [31]. In a post hoc subgroup analysis of the SOLOIST-WHF (sotagliflozin in patients with diabetes and recent worsening heart failure) trial, the primary endpoint was assessed according to the etiology of HF, comparing the drug effect in patients with ischemic versus non-ischemic HF, suggesting that the greatest benefit was observed in ischemic HF (*n* = 712 vs. 503; HR (95% CI) for sotagliflozin vs. placebo 0.55; 0.41, 0.74 vs. 0.88; 0.59, 1.31) [32]. Surprisingly, in the REVIVE I and II (effect of levosimendan on the short-term clinical course of patients with acutely decompensated heart failure), GALACTIC (effect of a strategy of comprehensive vasodilation vs. usual care on mortality and heart failure rehospitalization among patients with acute heart failure), and VICTORIA (vericiguat in patients with heart failure and reduced ejection fraction) trials, there are no data on differences in outcomes between ischemic vs. non-ischemic HF patients [33,34,35]. On the contrary, the LeoDOR (repetitive levosimendan infusions for patients with advanced chronic heart failure in the weakening post-discharge period) trial reports the percentages of patients with non-ischemic HF treated with levosimendan compared with placebo (ischemic HF 38.7% vs. 50%, *p* = 0.127; DCM 69.9% vs. 53.8%, *p* = 0.04; restrictive cardiomyopathy (RCM) 2.2% vs. 0% *p* = 0.410) [36]. In the subgroup analyses of the PUSH-AHF (natriuresis-guided diuretic therapy in acute heart failure: a pragmatic randomized trial) trial, the main findings (better natriuresis without significant benefit in clinical endpoints) were consistent across subgroups based on ischemic or non-ischemic etiology with negative interaction *p*-values for both dual primary endpoints (24 h urinary sodium excretion and a combined endpoint of time to all-cause mortality or adjudicated heart failure rehospitalization at 180 days) [37]. In the subgroup analysis of the PREMIER (in-hospital initiation of angiotensin receptor–neprilysin inhibition in acute heart failure) trial, the benefit of sacubitril/valsartan on NT-proBNP levels at week 8 was consistent in patients with ischemic and non-ischemic HF (*p* for interaction = 0.781) [38]. In a subgroup analysis of the UNLOAD (ultrafiltration versus intravenous diuretics for patients hospitalized for acute decompensated heart failure) trial, compared with intravenous diuretics, ultrafiltration resulted in lower HF rehospitalization rates, and this benefit seemed to be greater in non-ischemic HF patients than in ischemic HF patients (HR 0.21, CI: 0.05–1.00 vs. 0.72, CI: 0.33–1.56) [39]. In the CUORE (continuous ultrafiltration for congestive heart failure) trial, only the percentages of ischemic HF vs. non-ischemic HF patients in the control vs. ultrafiltration groups were reported (55% vs. 59%, *p* = 0.55; 17% vs. 15%, *p* = 0.81) [40].

Finererone, a non-steroidal mineralcorticoid receptor antagonist, is currently under investigation in the MOONRAKER program, including four randomized phase III clinical trials in HF settings, including AHF. In particular, the REDEFINE-HF (NCT06008197) trial is comparing finerenone vs. placebo in patients hospitalized for acute HF with HFmrEF and HFpEF, focusing on in-hospital initiation to improve CV outcomes. Moreover, CONFIRMATION-HF (NCT06024746) is assessing the combination of finerenone and an SGLT2 inhibitor, compared with standard care, in patients with acute HF, regardless of ejection fraction, to investigate the benefits of early, intensive combination therapy. Patients with known CMPs will be excluded from both trials.

Table 2 reports the details of the main randomized AHF trials related to CS. This is a peculiar setting characterized by critical patients with high rates of mortality. Most of the patients included in these trials present with CS related to MI and there are limited data specifically related to non-ischemic CS and to possible differences among the characteristics and outcomes according to different etiologies. Only the ECMO-CS (extracorporeal membrane oxygenation in the therapy of cardiogenic shock) trial reported the percentage of patients with DCM (10.9% in the VA-ECMO group vs. 15.5% in the conservative group, *p* = 0.471), but again, clinical outcomes according to CS etiology remain unknown [41].

**Table 1:** Main randomized trials of acute heart failure.

	Patients (*n*)	Year of Publication	Population Characteristics	Randomized Arms	Primary Endpoint	Main Results
SURVIVE trial [28]	1327	2007	Patients hospitalized with ADHF who required inotropic support	To receive intravenous levosimendan vs. dobutamine	All-cause mortality at 180 days	26% vs. 28% (HR 0.91; 95% CI, 0.74–1.13; *p* = 0.40)
UNLOAD trial [39]	200	2007	Patients hospitalized for HF with ≥2 signs of hypervolemia	To ultrafiltration or intravenous diuretics	(1) Weight loss at 48 h (2) Dyspnea assessment at 48 h	(1) 5.0 ± 3.1 kg vs. 3.1 ± 3.5 kg; *p* = 0.001) (2) Dyspnea scores (5.4 ± 1.1 [range 3 to 7] vs. 5.2 ± 1.2 [range 1 to 7]; *p* = 0.588)
CHAMPION trial [29]	550	2011	Patients with NYHA class III symptoms, irrespective of the LVEF and with a previous hospital admission for HF	To management with a W-IHM system (treatment group) or without (control group)	The rate of HF-related hospitalizations at 6 months	84 vs. 120 (rate 0.32 vs. 0.44, HR 0.72, 95% CI 0.60–0.85, *p* = 0.0002)
DOSE trial [42]	308	2011	ADHF patients	To receive furosemide administered intravenously by means of either a bolus every 12 h or continuous infusion and at either a low dose (equivalent to the patient’s previous oral dose) or a high dose (2.5 times the previous oral dose)	(1) Patients’ global assessment of symptoms, quantified as the AUC of the score on a visual analog scale over the course of 72 h (2) The change in the serum creatinine level from baseline to 72 h	(1) In the comparison of bolus with continuous infusion, patients’ global assessment of symptoms (mean AUC, 4236 ± 1440 vs. 4373 ± 1404, respectively; *p* = 0.47) or in the mean change in the creatinine level (0.05 ± 0.3 mg per deciliter [4.4 ± 26.5 μmol per liter] vs. 0.07 ± 0.3 mg per deciliter [6.2 ± 26.5 μmol per liter], respectively; *p* = 0.45). (2) In the comparison of the high-dose strategy with the low-dose strategy, patients’ global assessment of symptoms in the high-dose group (mean AUC, 4430 ± 1401 vs. 4171 ± 1436; *p* = 0.06). In the mean change in the creatinine level (0.08 ± 0.3 mg per deciliter [7.1 ± 26.5 μmol per liter] vs. 0.04 ± 0.3 mg per deciliter [3.5 ± 26.5 μmol per liter], *p* = 0.21)
CARRESS-HF trial [43]	188	2012	Patients with ADHF, worsened renal function (increase in serum creatitine >0.3 mg/dL from baseline), and signs and symptoms of persistent congestion	To a strategy of stepped pharmacologic therapy or ultrafiltration	The bivariate change from baseline in serum creatinine levels and body weight, as assessed 96 h after randomization	(1) At 96 h, the mean change in the creatinine level was −0.04 ± 0.53 mg per deciliter (−3.5 ± 46.9 μmol per liter) in the pharmacologic therapy group vs. +0.23 ± 0.70 mg per deciliter (20.3 ± 61.9 μmol per liter) in the ultrafiltration group (*p* = 0.003). (2) Difference in weight loss at 96 h in the pharmacologic therapy group vs. in the ultrafiltration group (a loss of 5.5 ± 5.1 kg [12.1 ± 11.3 lb] and 5.7 ± 3.9 kg [12.6 ± 8.5 lb], respectively; *p* = 0.58)
REVIVE I and II trials [30]	100 (I) + 600 (II)	2013	ADHF patients with LVEF < 35%	To receive intravenous levosimendan or placebo for 24 h in addition to standard treatment	A composite that evaluated changes in clinical status during the first 5 days after randomization (time points 6 h, 24 h, and 5 days)	58 patients in the levosimendan group vs. 82 in the placebo group experienced clinical worsening (*p* = 0.015 for the difference between the groups)
ASTRONAUT trial [25]	1615	2013	Patients with EF and LVEF of 40% or less, BNP ≥ 400 pg/mL or NT-proBNP ≥ 1600 pg/mL, and signs and symptoms of fluid overload	To receive 150 mg (increased to 300 mg as tolerated) of aliskiren or placebo daily, in addition to standard therapy	CV death or HF rehospitalization at 6 months and 12 months	24.9% of patients receiving aliskiren vs. 26.5% of patients receiving placebo at 6 months (HR, 0.92; 95% CI, 0.76–1.12; *p* = 0.41). At 12 months, the event rates were 35.0% for the aliskiren group vs. 37.3% for the placebo group (HR, 0.93; 95% CI, 0.79–1.09; *p* = 0.36)
ROSE trial [44]	360	2013	Hospitalized patients with AHF and renal dysfunction (eGFR of 15–60 mL/min/1.73 m^2^)	1:1 allocation ratio to the dopamine or nesiritide strategy. Within each strategy, participants were randomized in a double-blind, 2:1 ratio to active treatment or placebo	(1) 72 h cumulative urine volume (decongestion endpoint) (2) the change in serum cystatin C from enrollment to 72 h (renal function endpoint)	(1) Low-dose dopamine had no significant effect on 72 h cumulative urine volume vs. placebo (dopamine, 8524 mL; 95% CI, 7917–9131 vs. placebo, 8296 mL; 95% CI, 7762–8830; difference, 229 mL; 95% CI, −714 to 1171 mL; *p* = 0.59) or on the change in cystatin C level (dopamine, 0.12 mg/L; 95% CI, 0.06–0.18 vs. placebo, 0.11 mg/L; 95% CI, 0.06–0.16; difference, 0.01; 95% CI, −0.08 to 0.10; *p* = 0.72). (2) Low-dose nesiritide had no significant effect on 72 h cumulative urine volume vs. placebo (nesiritide, 8574 mL; 95% CI, 8014–9134 vs. placebo, 8296 mL; 95% CI, 7762–8830; difference, 279 mL; 95% CI, −618 to 1176 mL; *p* = 0.49) or on the change in cystatin C level (nesiritide, 0.07 mg/L; 95% CI, 0.01–0.13 vs. placebo, 0.11 mg/L; 95% CI, 0.06–0.16; difference, −0.04; 95% CI, −0.13 to 0.05; *p* = 0.36)
CUORE Trial [40]	56	2014	Patients with severe congestive HF	To receive standard medical therapy or ultrafiltration as first-line treatment	Rehospitalizations for congestive HF during 1 year of FU	A lower incidence of rehospitalizations for HF in the ultrafiltration-treated patients (HR:0.14, 95% CI 0.04–0.48; *p* = 0.002)
AVOID-HF trial [45]	224	2015	Patients hospitalized for congested HF	To AUF or ALD	First HF event within 90 days after hospital discharge	Estimated days to first HF event for the AUF and ALD group are, respectively, 62 vs. 34 (*p* = 0.106)
BLAST-AHF trial [46]	621	2017	Patients hospitalized with AHF, as evidenced by elevated natriuretic peptides and at least two physical HF signs including congestion on chest radiograph, rales, edema, and/or elevated jugular venous pressure	To randomly assigned to one of the following four (1:1:1:1: placebo, 1, 5, or 25 mg/h of TRV027 for at least 48 h and up to 96 h)	(1) Time from baseline to death through day 30 (2) Time from baseline to HF rehospitalization through day 30 (3) The first assessment time point following worsening of HF through day 5 (4) Change in dyspnea VAS score calculated as AUC representing the change from baseline over time through day 5 (5) The length of initial hospital stays (in days) from baseline	No significant differences were observed between any of the dose groups compared with placebo with regard to the primary endpoint
TRUE-AHF trial [30]	2157	2017	AHF patients defined as: an unplanned emergency department visit or hospitalization for AHF, dyspnea at rest that had worsened during the previous week, evidence of HF on chest radiography, a blood BNP level of more than 500 pg per milliliter, or an NT-proBNP level of more than 2000 pg per milliliter. All patients that continued to have dyspnea at rest for at least 2 h after IV furosemide at a dose of 40 mg (or equivalent) with sBP between 116–180 mmHg	To receive a continuous intravenous infusion of either Ularitide at a dose of 15 ng per kilogram of body weight per minute or matching placebo for 48 h, in addition to accepted therapy	Death from CV causes during a median follow-up of 15 months and clinical composite endpoint during the first 48 h	236 vs. 225 (21.7% vs. 21.0%; HR: 1.03; 96% CI, 0.85 to 1.25; *p* = 0.75). No change in clinical course.
PIONEER trial [27]	881	2019	Patients with HF with reduced EF who were hospitalized for ADHF (NT-proBNP concentration of 1600 pg per milliliter or more or a BNP) concentration of 400 pg per milliliter or more)	To receive sacubitril–valsartan vs. enalapril	The time-averaged proportional change in NT-pro-BNP concentration from baseline through weeks 4 and 8	0.53 in the sacubitril–valsartan group vs. 0.75 in the enalapril group (percent change, −46.7% vs. −25.3%; ratio of change with sacubitril–valsartan vs. enalapril, 0.71; 95% CI, 0.63 to 0.81; *p* < 0.001)
RELAX AHF 2 trial [31]	6545	2019	Patients with AHF and dyspnea, vascular congestion on chest radiography, increased BNP, mild-to-moderate renal insufficiency, and a systolic blood pressure of at least 125 mmHg	To receive either a 48 h intravenous infusion of serelaxin or placebo, in addition to standard care	(1) Death from CV causes at 180 days (2) Worsening HF at 5 days	(1) 8.7% vs. 8.9% (HR: 0.98; 95% CI: 0.83 to 1.15; *p* = 0.77) (2) 6.9% vs. 7.7% (HR: 0.89; 95% CI, 0.75 to 1.07; *p* = 0.19)
GALACTIC trial [34]	781	2019	AHF patients with dyspnea, increased plasma concentrations of natriuretic peptides, and systolic blood pressure of at least 100 mm Hg	To a strategy of early intensive and sustained vasodilation (individualized doses of sublingual and transdermal nitrates, low-dose oral hydralazine for 48 h, and rapid up-titration of angiotensin-converting enzyme inhibitors, angiotensin receptor blockers, or sacubitril-valsartan) vs. usual care	Composite of all-cause mortality or rehospitalization for AHF at 180 days	30.6 % vs. 27.8% (absolute difference for the primary endpoint, 2.8% [95% CI, −3.7% to 9.3%]; adjusted HR: 1.07 [95% CI, 0.83–1.39]; *p* = 0.59)
AFFIRM-AHF trial [24]	1108	2020	AHF with concomitant iron deficiency (defined as ferritin < 100 μg/L, or 100–299 μg/L with transferrin saturation < 20%) and LVEF < 50%	To receive intravenous ferric carboxymaltose or placebo for up to 24 weeks, dosed according to the extent of iron deficiency	A composite of total hospitalizations for HF and CV death up to 52 weeks after randomization	32% vs. 38% of patients in the placebo group (HR 0.80, 95% CI 0.66–0.98, *p* = 0.030)
VICTORIA trial [35]	5050	2020	Patients with HF and LVEF > 45% who had recently been hospitalized or had received intravenous diuretic therapy	To receive vericiguat (target dose, 10 mg once daily) or placebo, in addition to guideline-based medical therapy	A composite of death from CV causes or first hospitalization for HF	37.9% vs. 40.9% in the placebo group (HR: 0.90; 95% CI, 0.83 to 0.98; *p* = 0.02)
Sean P. Collins et al. trial [47]	479	2020	AHF patients with a history of HF and a discharge within 23 h or less	To usual care vs. a tailored self-care intervention	A global rank of CV death, HF-related events (unscheduled clinic visit due to HF, ED revisit, or hospitalization), and changes in the KCCQ-12 summary score at 90 days	No significant difference in the primary outcome between patients in the intervention vs. usual care arms ([HR], 0.89; 95% CI, 0.73–1.10; *p* = 0.028)
SOLOIST-WHF trial [32]	1222	2021	Patients with type 2 diabetes mellitus who were recently hospitalized for worsening HF	To receive sotagliflozin or placebo	The total number of deaths from CV causes and hospitalizations and urgent visits for HF	51.0 vs. 76.3 (HR: 0.67; 95% CI: 0.52 to 0.85; *p* < 0.001)
ADVOR trial [20]	519	2022	ADHF patients with clinically evident volume overload, elevated NT-pro-BNP, and loop diuretic therapy for at least 1 month before randomization	To receive either intravenous acetazolamide vs. placebo added to standardized intravenous loop diuretics	Successful decongestion assessed by a dedicated score indicating no more than trace edema within 3 days after randomization	42.2% vs. 30.5% (*p* < 0.001)
STRONG-HF trial [26]	1078	2022	Patients with AHF not treated with full doses of guideline-directed drug treatment	Rapid up-titration of treatments before discharge vs. standard approach	180-day readmission to hospital due to HF or all-cause death	15.2% vs. 23.3% (adjusted risk difference 8.1% [95% CI 2.9–13.2]; *p* = 0.0021; risk ratio 0.66 [95% CI 0.50–0.86])
EMPULSE trial [23]	530	2022	Primary diagnosis of AHF or ADHF regardless of LVEF, randomized when clinically stable (after 3 days)	To receive empagliflozin 10 mg once daily or placebo for 90 days	Clinical benefit, defined as a hierarchical composite of death from any cause, number of heart failure events and time to first heart failure event, or a 5 point or greater difference in change from baseline in the Kansas City Cardiomyopathy Questionnaire Total Symptom Score at 90 days	More patients treated with empagliflozin displayed a clinical benefit compared with placebo (stratified win ratio, 1.36; 95% confidence interval, 1.09–1.68; *p* = 0.0054), meeting the primary endpoint.
CLOROTIC trial [21]	230	2023	Patients with CHF treated with oral furosemide (80–240 mg/day) for 1 month before admission for AHF	To receive HCTZ vs. placebo in addition to an intravenous furosemide regimen	Changes in: (1) Body weight (2) Patient-reported dyspnea 72 h after randomization	(1) HCTZ group vs. placebo group: −2.3 vs. −1.5 kg; (95% CI: −1.14 (−1.84 to −0.42); *p* = 0.002) (2) HCTZ group vs. placebo group: AUC: 960 vs. 720; *p* = 0.497
COACH trial [22]	5452	2023	Patients with AHF who were seeking emergency care	In the intervention phase, low-risk patients (according to a point-of-care algorithm) were discharged early (in ≤3 days) vs. high-risk patients, who were admitted to the hospital	A composite of death from any cause or hospitalization for CV causes within: (1) 30 days after presentation (2) 20 months	(1) Within 30 days, 12.1% patients enrolled during the intervention phase vs. 14.5% in the control phase (adjusted HR: 0.88; 95% CI, 0.78 to 0.99; *p* = 0.04) (2) Within 20 months, 54.4% in the intervention phase vs. 56.2% in the control phase (adjusted HR: 0.95; 95% CI, 0.92 to 0.99)
LeoDOR trial [36]	148	2023	AHF patients requiring IV diuretics, IV inotropic therapy, IV vasodilators, or any combination of these. LVEF < 30%. At least one hospitalization or clinic visit for HF within 12 months before admission. NTproBNP elevated or NYHA III-IV symptoms after stabilization.	Patients were randomly assigned (2:1) to receive either levosimendan or placebo	A global rank endpoint in which all participants, regardless of treatment assignment, were ranked across three hierarchical tiers: Tier 1 = time to death or urgent heart transplantation or implantation of a ventricular assist device (VAD). Tier 2 = time to non-fatal HF requiring IV vasoactive therapy. Tier 3 = time-averaged proportional change in NT-proBNP from baseline to week 14, each multiplied by the number of elapsed times in weeks, divided by the total number of 14 weeks of observation from baseline to 14 weeks.	The mean rank score was 72.55 for the levosimendan group vs. 73.81 for the placebo group (*p* = 0.863)
PUSH-AHF trial [37]	310	2023	AHF patients requiring treatment with intravenous loop diuretics	To receive natriuresis-guided therapy or standard of care	The dual primary endpoints are: (1) 24 h urinary sodium excretion and (2) A combined endpoint of time to all-cause mortality or adjudicated HF rehospitalization at 180 days	(1) 24 h urinary sodium excretion is 409 ± 178 mmol in the natriuresis-guided therapy group versus 345 ± 202 mmol in the control group, respectively (*p* = 0.0061) (2) Combined endpoint of time to all-cause mortality or first HF rehospitalization occurred in 31% and 31% of patients in the natriuresis-guided and control arms, respectively (HR: 0.92 (3) [95% CI 0.62–1.38], *p* = 0.6980)
SSU-AHF trial [48]	193	2024	Lower-risk patients who presented to the ED with signs and symptoms of AHF	To SSU or hospital admission from the ED	(1) DAOOH at 30-day follow-up (2) Quality of life measured as KCCQ-12	(1) 1.6 more DAOOH at 30-day follow-up in the SSU arm vs. those in the hospitalization arm (median [IQR], 26.9 [24.4–28.8] vs. 25.4 [22.0–27.7] days; *p* = 0.02) (2) The mean (SD) KCCQ-12 summary score between the SSU and hospitalization arms is 51.3 (25.7) vs. 45.8 (23.8) points, respectively (*p* = 0.19)
COLICA trial [49]	278	2024	AHF patients with evidence of congestion requiring at least 40 mg of intravenous (i.v.) furosemide and elevated concentrations of NT-proBNP > 900 pg/mL	To receive either colchicine (loading dose 2 mg, followed by 0.5 mg every 12 h for 8 weeks) or placebo within the first 24 h of presentation	The time-averaged proportional change in NT-pro-BNP concentration from baseline through weeks 4 and 8	Colchicine group [−62.2%, 95% CI −68.9% to −54.2%] vs. the placebo group (−62.1%, 95% CI −68.6% to −54.3%)
PREMIER trial [38]	400	2024	In patients stabilized after hospitalization for AHF irrespective of LVEF	To continue angiotensin-converting enzyme inhibitor or angiotensin receptor blocker (control group) or to switch to Sac/Val (Sac/Val group)	8-week proportional change in geometric means of NT-pro-BNP levels	The percent changes in NT-pro-BNP level geometric means at weeks 4/8 are −35%/−45% (Sac/Val group) and −18%/−32% (control group), and their group ratio (Sac/Val vs. control) is 0.80 (95% CI 0.68–0.94; *p* = 0.008) at week 4 and 0.81 (95% CI 0.68–0.95; *p* = 0.012) at week 8, respectively
DICTATE-AHF trial [50]	240	2024	Hypervolemic hospitalized AHF	To dapagliflozin 10 mg once daily or structured usual care with protocolized diuretic titration until day 5 or hospital discharge	Diuretic efficiency expressed as cumulative weight change per cumulative loop diuretic dose	No difference between dapagliflozin vs. usual care in diuretic efficiency (OR: 0.65; 95% CI: 0.41–1.02; *p* = 0.06)

ADHF: acutely decompensated heart failure, AHF: acute heart failure; ALD: adjustable intravenous loop diuretics; UC: area under the curve; AUF: adjustable ultrafiltration; BNP: B-type natriuretic peptide; CHF: chronic heart failure; CI: confidence interval; CV: cardiovascular; DAOOH: days alive and out of hospital; ED: emergency department; eGFR: estimated glomerular filtration rate; HCTZ: hydrochlorothiazide; HF: heart failure; HR: hazard ratio; LVEF: left ventricle ejection fraction; KCCQ-12: 12-item Kansas City Cardiomyopathy Questionnaire; NYHA: New York Heart Association; NT-pro-BNP: N-terminal pro-B-type natriuretic peptide; W-IHM: wireless implantable hemodynamic monitoring; OR: odds ratio; SSU: short-stay units; VAS: visual analog scale; TRV027: a biased ligand of the angiotensin II type 1 receptor (AT1R).

**Table 2:** Main recent randomized trials on cardiogenic shock.

	Patients (*n*)	Year of Publication	Population Characteristics	Randomized Arms	Primary Endpoint	Main Results
ISAR-SHOCK trial [51]	25	2008	AMI patients with CS	To receive Impella LP 2.5 (Abiomed Europe GmbH, Aachen, Germany) or IABP	Change in the CI from baseline to 30 min after implantation	Impella: ΔCI: 0.49 ± 0.46 l/min/m^2^; IABP: ΔCI: 0.11± 0.31 l/min/m^2^; *p* = 0.02
Joerg T. Fuhrmann et al. trial [52]	32	2008	Persistent refractory CS (systolic blood pressure < 90 mmHg or requirement of inotropic amines and vasopressors to maintain an unaugmented systolic blood pressure of at least 90 mm Hg, a CI below 2.5 L/min/m^2^, a PCOP above 18 mm Hg, and clinical signs of hypoperfusion) within 2 h after PCI	Infusion of either levosimendan or enoximone after initiation of current therapy, always including revascularization, intra-aortic balloon pump counterpulsation, and inotropes	All-cause mortality at 30 days	Survival rate at 30 days in the levosimendan-treated group (69%) vs. enoximone group (37%, *p* = 0.023)
IABP SHOCK I trial [53]	45	2010	Patients undergoing PCI for AMI with CS	To receive IABP or not	APACHE II score after 4 days	In the IABP group, the APACHE II score was 18.2 ± 3.7 vs. 20.0 ± 2.4 in the standard treatment group. Hospital mortality was 36.8% in the IABP group vs. 28.6% in the standard treatment group.
SOAP II trial [54]	1679	2010	Patients with CS, defined as mean arterial pressure less than 70 mmHg or systolic blood pressure less than 100 mm Hg, despite an adequate amount of fluids, and with signs of tissue hypoperfusion	To receive either dopamine or norepinephrine as first-line vasopressor therapy to restore and maintain blood pressure	The rate of death at 28 days after randomization	Rate of death at 28 days (52.5% in the dopamine group and 48.5% in the norepinephrine group; odds ratio with dopamine: 1.17; 95% CI: 0.97 to 1.42; *p* = 0.10)
IABP-SHOCK II trial [55]	600	2012	Patients undergoing revascularization for AMI with CS	To IABP or no IABP	30-day all-cause mortality	39.7% in the IABP group vs. 41.3% in the control group (RR with IABP, 0.96; 95% CI, 0.79 to 1.17; *p* = 0.69)
IMPRESS shock trial [56]	48	2017	AMI patients with severe CS undergoing immediate revascularization	To receive Impella CP or IABP	30-day all-cause mortality	Mortality in patients treated with either IABP or Impella CP was similar (50% and 46%, respectively; HR with Impella CP: 0.96; 95% CI: 0.42 to 2.18; *p* = 0.92)
Bruno Levy et al. trial [57]	57	2018	CS due to successfully revascularized AMI	To receive epinephrine or norepinephrine	(1) The change in cardiac index (2) The incidence of refractory CS	(1) Cardiac index evolution was similar between the 2 groups (*p*: 0.43) (2) Higher incidence of refractory shock in the epinephrine group (37% vs. 7% in the norepinephrine group; *p* = 0.008)
IMPELLA-STIC trial [58]	12	2019	Patients with AMI complicated by CS	To receive IABP vs. Impella LP 5.0 + IABP	Change in CPI from baseline to 12 h after implantation, measured with a Swan–Ganz catheter	IABP group: CPI = 0.08 ± 0.08W/m^2^ Impella LP5.0 + IABP group: CPI = −0.02 ± 0.25 W/m^2^; *p* = 0.4
ECMO-CS trial [41]	117	2022	Patients with either rapidly deteriorating or severe CS	To immediate VA-ECMO or non-immediate VA-ECMO	The composite of death from any cause, resuscitated circulatory arrest, and implementation of another mechanical circulatory support device at 30 days	63.8% of patients in the immediate VA-ECMO group vs. 71.2% of patients in the non-immediate VA-ECMO group, respectively (HR: 0.72 [95% CI, 0.46–1.12]; *p* = 0.21)
ECLS-SHOCK trial [59]	420	2023	Patients with AMI complicated by CS	To receive early ECLS plus usual medical treatment or usual medical treatment alone	Death from any cause at 30 days	47.8% in the ECLS group vs. 49.0% in the control group (RR: 0.98; 95% CI: 0.80 to 1.19; *p* = 0.81)
EURO SHOCK trial [60]	35	2023	AMI patients with persistent CS 30 min after PPCI of the culprit lesion	To receive either VA-ECMO or continue with standard therapy	30-day all-cause mortality	43.8% of patients in the VA-ECMO group vs. 61.1% of patients in standard therapy (HR: 0.56, 95% CI: 0.21–1.45; *p* = 0.22)
DanGer Shock trial [61]	355	2024	Patients with AMI complicated by CS	To receive Impella CP plus standard care or standard care alone	Death from any cause at 180 days	45.8% in the Impella group vs. 58.5% in the standard care group (HR: 0.74; 95% CI: 0.55 to 0.99; *p* = 0.04)

AMI: acute myocardial infarction; APACHE: Acute Physiology and Chronic Health Evaluation (APACHE); PI: cardiac power index; CI: confidence interval; CS: cardiogenic shock; HR: hazard ratio; IABP: intra-aortic balloon pump; PCOP: post-Capillary Pulmonary Hypertension; PPCI: primary percutaneous coronary intervention; RR: relative risk; VA-ECMO: venoarterial extracorporeal membrane oxygenation.

### 2.6. Evidence on ADHF Rates in Randomized Trials of Chronic HF

Table 3 reports the main randomized trials on chronic HF, with a focus on the rate of ADHF during follow-up. However, again, the different etiologies of patients included in the trials are often unreported. Among DAPA-HF (dapagliflozin and prevention of adverse outcomes in heart failure) trial patients (*n* = 4744), 1687 had non-ischemic CMP, but the rates of idiopathic DCM or other types of CMPs, especially the differential impact of etiology on the hospitalization rate, are unknown [62]. In the subgroup analysis of the EMPEROR reduced (effect of empagliflozin on the clinical stability of patients with heart failure and a reduced ejection fraction) trial, the benefit from the addition of empagliflozin was consistent in patients with both ischemic and non-ischemic HF [63]. In the IRONMAN (intravenous ferric derisomaltose in patients with heart failure and iron deficiency) trial, the percentages of ischemic and non-ischemic HF patients are reported (58% vs. 56% in the ferric derisomaltose group; 31% vs. 35% in the usual care group), but without specific details on CMP type or outcomes [64]. The TRANSFORM-HF (effect of torsemide vs. furosemide after discharge on all-cause mortality in patients hospitalized with heart failure) trial only reported the percentages of ischemic HF patients (29.8% in the torsemide group vs. 26.7% in the furosemide group) [65]. Similarly, in the DIAMOND (patiromer for the management of hyperkalemia in heart failure with reduced ejection fraction) trial, ischemic HF patients made up 72.2% of the patiromer group vs. 70.6% of the placebo group [66]. In the PARADIGM-HF (angiotensin–neprilysin inhibition versus enalapril in heart failure) trial, ischemic HF patients made up 59.9% of the angiotensin receptor–neprilysin inhibitor group vs. 60.1% of the enalapril group [67]. Conversely, the subgroup analysis of the primary efficacy endpoint of the SOLOIST-WHF (sotagliflozin in patients with diabetes and recent worsening heart failure) trial showed a consistent treatment effect across the subgroups stratified according to ischemic vs. non-ischemic HF etiology [32]. Finally, the recent RESHAPE-HF2 (randomized investigation of the MitraClip device in heart failure: second trial in patients with clinically significant functional mitral regurgitation) and MATTERHORN (transcatheter repair versus mitral valve surgery for secondary mitral regurgitation) trials, although providing important evidence on the effectiveness of mitral transcatheter edge-to-edge repair (M-TEER) in symptomatic HF with functional mitral regurgitation, did not specifically analyze the outcomes based on ischemic or non-ischemic etiology [68,69].

## 3. AHF and Non-Ischemic Cardiomyopathies

CMPs are a heterogeneous group of myocardial disorders in which the heart muscle is structurally and functionally abnormal in the absence of CAD, hypertension, valvular disease, and congenital heart disease (CHD), leading to the observed myocardial disease [75]. The current European guidelines on CMPs identify five main clinical phenotypes: DCM, HCM, RCM, NDLVC, and ARVC [75]. CMPs may be inherited (genetic/familial) or acquired and represent one of the main causes of HF in young populations [76,77,78]. However, specific data on patients with CMPs that evolve into AHF are limited. As mentioned above, HF studies often omit the characterization of HF etiology, and when reported, a generic distinction between ischemic and non-ischemic groups is made. A study based on data from the ESC Heart Failure registries was designed to evaluate the prevalence, clinical characteristics, management, and outcomes of patients with two main etiologies of HF (ischemic and non-ischemic CMPs) and showed that ischemic CMP patients were older and had more comorbidities, while non-ischemic patients had worse systolic EFs. There were no other differences in terms of using HF guideline-recommended medications, implantable cardioverter defibrillators (ICDs), or cardiac resynchronization therapy. Furthermore, all-cause death, as well as all-cause death and readmissions for HF at 12 months, occurred more frequently in the ischemic group compared with the non-ischemic group (15.9% vs. 10%, *p* = 0.016; and 40.9% vs. 28.6%, *p* = 0.00089, respectively) [79]. Cardiac magnetic resonance (CMR) is crucial in these patients. A study of 168 patients assessed the role of late gadolinium enhancement (LGE) between ischemic and non-ischemic CMP patients and demonstrated that in ischemic patients, LGE extent (≥median) and inferior wall LGE independently predicted the primary endpoint (ventricular tachycardia (VT)). In non-ischemic patients, LGE extent (≥median, number of LGE segments, LGE stratified per 5% increase) and mid-wall LGE were independent predictors of the primary endpoint. Therefore, LGE extent, location, and pattern characteristics were greater predictors of worse outcomes in patients with non-ischemic rather than ischemic CMP [80].

### 3.1. Dilated Cardiomyopathy

DCM is defined by the presence of LV dilatation and systolic dysfunction unexplained solely by abnormal loading conditions or CAD [75]. The estimated prevalence is 0.036–0.4% [81,82]. DCM may be inherited in 30–50% of cases. In another 40% of cases, it is caused by genetic mutations in sarcomere proteins, the cytoskeleton, the nuclear envelope, sarcolemma, ion channels, and/or intercellular junction molecules or acquired due to infection, exposure to toxic agents, endocrine or metabolic pathologies, or peripartum conditions [75,83,84]. As regards the genetic form, most of the mutations have an autosomal dominant inheritance transmission. A truncating titin mutation is the most frequent mutation (13% and 25% of non-familial and familial cases of DCM, respectively [85]) and desmoplakin (DSP), filamin C (FLCN), lamin A/C (LMNA), phospholamban (PLN), and RNA binding motif protein 20 (RMB20) mutations are strong predictors of sudden cardiac death (SCD) [75].

#### 3.1.1. AHF in DCM

In patients with DCM, advanced HF represents the most frequent cause of death, while SCD affects only 30% of patients [86,87]. Determining the incidence and prevalence of AHF in DCM is challenging because HF etiology is under-reported in clinical trials and observational studies, as mentioned above. When the etiology of HF is reported, it is mainly described as ischemic or non-ischemic, but what the latter group includes often remains unknown. In the ESC-HF-LT registry, a prospective observational study that collected hospitalization and 1-year follow-up data from 6629 AHF patients, 13.6% had DCM, and among these, the most common presentation of AHF was CS or ADHF (18.4% vs. 18.5%, respectively) [3]. In a retrospective observational study on patients admitted for AHF to intensive care units (ICUs), in which the aim was to investigate the prevalence of acute kidney injury (AKI), many patients were affected by idiopathic DCM (47%) [88]. In a multicenter randomized trial that compared the use of dopamine and norepinephrine in the treatment of CS, out of a total of 1679 patients, only 44 patients had DCM [54], while in an open-label randomized trial of 85 patients admitted to the ICU for CS, eight had DCM [89]. However, none of these studies report the outcome of patients affected by AHF due to DCM. A meta-analysis of three trials (DOSE [diuretic strategies in patients with acute decompensated heart failure] [42], ROSE [acute heart failure randomized trial] [44], and CARRESS-HF [ultrafiltration in decompensated heart failure with cardiorenal syndrome [43]) evaluated the progression to AHF in ischemic vs. non-ischemic patients. Among 762 patients, 43.1% had a history of non-ischemic CMP and these patients had a similar course of decongestion, self-assessment of well-being, and dyspnea, with no significant differences in 60-day composite all-cause mortality or hospitalization for HF compared to ischemic patients. However, non-ischemic patients with LVEFs < 40% scored more highly regarding their global sense of well-being (global visual analog scale; +25.5 vs. +19.1, *p* = 0.023) and improvement in serum creatinine (0.031 mg/100 mL vs. +0.042 mg/100 mL, *p* = 0.009) at 72 h (Table 1) [90]. Different outcomes were found by Cherbi et al. in the prospective FRENSHOCK registry, which includes 772 CS patients and analyzes the 1-year outcomes (rehospitalizations, mortality, heart transplants (HTs), left ventricular assist devices (LVADs)) of idiopathic DCM patients (10.1%). Among patients with CS, idiopathic DCM was a very common scenario, with higher rates of 1-year death or CV rehospitalizations (adjusted odds ratio of 4.77 [95% CI 1.13 to 20.1], *p* = 0.03) and higher rates of HT or LVAD for patients aged < 65 years (adjusted odds ratio of 2.68 [1.21 to 5.91], *p* = 0.02) [91]. Furthermore, in the same registry, the authors evaluated the prevalence of potential ventricular arrhythmias (VA) as a trigger of CS, demonstrating that in the VA-triggered CS group (12.2%), there was no 1-year mortality difference between ischemic and non-ischemic DCMs (42.5 vs. 42.6%, HR 0.97 (0.52–1.81), *p* = 0.92). However, idiopathic DCMs led to higher rates of HT and LVAD implantation (25.9 vs. 5%, *p* = 0.02) [92]. Meanwhile, Miric et al. demonstrated that, among patients hospitalized for AHF, patients with CMD had more severe RV dysfunction, measured by RV free wall strain, compared to ischemic patients with the same functional class and outcome [93]. DCM patients with advanced HF may have three different clinical evolutions: (1) structural and functional recovery following an incident of HF, (2) remission of HF symptoms and stabilization of the LVEF, or (3) progression to HT (Figure 1) [94]. In recent years, outcomes have been improved by advances in HF treatment. In a cohort of 373 DCM patients, after guideline-directed medical therapy (GDMT), transplant-free survival at 1, 2, and 4 years of follow-up was 94%, 92%, and 88%, respectively, and survival free from HF hospitalization was 88%, 82%, and 78%, respectively [95]. Importantly, the continuation of HF treatment is crucial in patients who have recovered from DCM; the TRED-HF (withdrawal of pharmacological treatment for heart failure in patients recovered from dilated cardiomyopathy) trial showed that the withdrawal of GDMT for HF was associated with a 40% relapse of LV dysfunction within 6 months [96]. Furthermore, non-ischemic HF etiology has been reported as an independent predictor of LV functional recovery in a cohort of 3994 HF patients [97]. On the contrary, male sex, age > 60 years, Black race, lower baseline LVEF, higher New York Heart Association functional classification (NYHA) class, significant mitral regurgitation, and higher natriuretic peptide levels have been associated with adverse outcomes in non-ischemic DCM patients [86,95,98]. Furthermore, extended mid-wall myocardial LGE on CMR in DCM patients is associated with higher HF mortality and hospitalizations [87]. CMR is also important for recognizing “hot phases” of CMPs (also in HCM, ARCV, and NDLVC), characterized by worsening clinical status, including evolution into AHF or life-threatening arrhythmias. Indeed, in these patients, the presence of specific abnormalities on CMR, such as myocardial edema and an LGE with a ring-like pattern (involving mostly LV myocardial segments), has been associated with a worse prognosis [99].

#### 3.1.2. Diagnosis of DCM

The essential elements of the diagnostic work-up for DCM patients include clinical and family history, laboratory tests, ECGs, Holter monitoring, cardiac imaging, and genetic testing. Echocardiography is the cornerstone for DCM diagnosis, as it provides an overall evaluation of LV anatomy and function, associated valvular disease, atrial and RV function, and pulmonary pressure [100]. Nowadays, CMR has even greater value for two main reasons: (1) the distribution and extent of LGE has prognostic value for predicting arrhythmic risk and the severity of HF [101,102], and (2) it provides additional information on tissue characterization, generating etiological hypotheses (e.g., the presence of myocardial edema may suggest myocarditis or an inflammatory cause, while LGE distribution may exclude MI or generate etiological hypotheses such as subepicardial distribution in post-myocarditis forms, patchy in sarcoidosis, inferolateral in dystrophinopathies, septal mid-wall in LMNA carriers, ring-like in DSP and FLNC-truncating variant carriers, and iron deposition in hemochromatosis) [100,103].

#### 3.1.3. Treatment of DCM

In patients with HF and DCM, treatment should be guided by HF guidelines, while in AHF, in-hospital treatment with intravenous diuretics, vasodilators, or inotropes may be required, although there is no evidence of improved outcomes with these drugs [1]. Primary prevention ICD implantation is currently recommended in DCM patients with HF (NYHA class II–III) and an LVEF ≤ 35% on GDMT [1,75]. However, the DANISH (Danish study to assess the efficacy of ICDs in patients with non-ischemic systolic heart failure on mortality) trial failed to demonstrate a mortality benefit of primary prevention ICD implantation in patients with non-ischemic DCM and an EF < 35%, suggesting that more detailed and personalized stratification (accounting for etiology, LGE distribution, or high-risk genes such as DSP, FLCN, LMNA, PLN, and RMB) may be necessary in order to identify a subgroup of patients at a high risk of SCD who may benefit from primary prevention ICD implantation regardless of their LVEF [104,105,106]. Current knowledge facilitates therapies that can ameliorate the clinical consequences of causative gene mutations in some patients with DCM. For example, LMNA mutations are known to predispose individuals to VAs and SCD, regardless of LV function [107], while patients with PLN and RBM20 mutations should allow for more intensive follow-up and the timely provision of advanced HF therapies [75]. Accordingly, Paldino et al. demonstrated that genotypic-based classification showed higher precision in predicting the outcome of patients with CMP than phenotype-based classification. In particular, genotype-based classification, but not phenotype-based classification, was predictive of SCD/VAs, and LMNA showed the worst trends in terms of death/HT or death for HF/HT/LVAD [108]. In the future, genetic mutations may be targets for innovative therapeutic interventions.

#### 3.1.4. MCS and HT in DCM

Recently, the proportion of DCM patients with AHF requiring HT increased compared with other HF etiologies, and DCM is the third most common indication for heart and lung transplantation in adults [109,110,111,112]. In young and middle-aged adults, 64% and 51% of HT cases, respectively, are attributed to DCM [111], while, among older adults, DCM remains the second most common indication for HT after CAD. Similarly, >40% of DCM patients receive MCS, either as a bridge to HT or as a destination therapy [113,114]. Regarding the prognosis, favorable outcomes (with a median survival of 12.2 years) have been demonstrated after HT in patients with DCM [109,110,111]. Few data are available for DCM patients with AHF who are receiving LVAD. In a contemporary analysis of 24,809 adults listed for HT, 2.7% of genetic DCM patients were less frequently delisted for clinical deterioration or death and more likely to be transplanted compared with those with non-ischemic DCM [HR: 0.617, 95% CI: 0.47–0.81; HR: 1.25, 95%CI: 1.14–1.37, respectively], perhaps because they were younger and without CAD and related organ dysfunction [115,116].

#### 3.1.5. Prognosis of DCM

Despite the advances made in pharmacological and interventional management, AHF remains the most frequent cause of death in DCM, overtaking mortality in other non-ischemic HF etiologies [86,87]. Furthermore, non-cardiac mortality should not be neglected in these patients, as approximately one-third of patients die of cancer, infections, pulmonary disease, or hemorrhage [86].

### 3.2. Hypertrophic Cardiomyopathy

HCM is defined by the presence of increased LV wall thickness (≥15 mm in adults and ≥13 mm in adults with first-degree relatives with HCM) or mass that is not solely explained by abnormal loading conditions. Approximately 40–70% of patients have obstructive HCM, diagnosed by an LV outflow tract (LVOT) gradient ≥30 mmHg at rest or during exercise [75,117,118,119]. HCM is the most common inherited heart disease among young adults, with a prevalence of 0.2% and 0.029% in adults and children, respectively, and is caused by genetic mutations affecting sarcomere genes in more than half of cases. These mutations are inherited in an autosomal dominant pattern [75]. Between 5 and 10% of cases of HCM may be caused by other etiologies: hereditary syndromes, neuromuscular disorders, glycogen and lysosomal storage diseases (e.g., Fabry, Pompe, or Danon disease), or mitochondrial disorders (the so-called phenocopies). In the remaining 25–30% of cases, the etiology is unknown.

#### 3.2.1. AHF in HCM

Patients with advanced HCM may present with two clinical phenotypes:

(1) The hyperkinetic-restrictive form is characterized by a small, stiff, and hypertrophic LV with severe diastolic dysfunction and a normal ejection fraction. In this case, HF presents as heart failure with a preserved ejection fraction (HfpEF) phenotype [120].
(2) The hypokinetic-dilated form is characterized by an increase in volume and a reduction in LV wall thickness. This clinical presentation may be similar to DCM and the differential diagnosis may be challenging in the absence of prior documentation of asymmetrical LV hypertrophy or a family history. Patients with this phenotype, characterized by fibrotic replacement, cardiomyocyte necrosis, and microvascular ischemia, develop AHF more frequently [121]. Furthermore, patients with mutations in thin-filament genes (TNNT2, TNNI3, TPM1, and ACTC) or multiple gene mutations in sarcomeric proteins are more susceptible to progression towards AHF compared with other mutations [122].

The prevalence of HF In patients with HCM is around 67%, and it is more prevalent in patients with obstructive HCM compared with non-obstructive HCM (only 10%) [123]. This is because pressure overload caused by dynamic LVOT obstruction during systole exacerbates the severity of HF [124]. However, the progression to AHF is infrequent and occurs only in 3% to 15% of HCM patients with an incidence ranging from 0.5% to 1.5% per year due to severe LV obstruction and hypertrophy or severe LV systolic dysfunction [121,125]. However, these data may be underestimated since patients with HCM are under-represented in AHF studies.

#### 3.2.2. Diagnosis of HCM

Multimodality imaging provides a comprehensive characterization for HCM diagnosis. The evaluation of LV wall thickness, LVEF estimation, the definition of LV apical aneurysms, LVOT obstruction, and the extent of LGE is essential for defining the diagnosis and the arrhythmic risk of HCM, as well as for predicting the progression to AHF. Echocardiography is essential for diagnosing the hypokinetic-dilated phenotype, reported in patients with end-stage HCM, characterized by regression of hypertrophy, LV spherical remodeling, and volume increase. The diagnostic work-up continues with a systematic approach that includes echocardiography during a Valsalva maneuver in the sitting, semi-supine, or standing positions to identify LVOT obstruction. Indeed, a gradient ≥30 mmHg at rest independently predicts HF progression and mortality [126], with progression to advanced HF in 20% of patients with severe LVOT obstruction [127]. Furthermore, exercise stress echocardiography is recommended in symptomatic patients without LVOT obstruction at rest [128], since induced exercise LVOT obstruction is associated with an increased risk of symptom onset at follow-up (3.2% vs. 1.6% per year for non-obstructive patients) [129]. CMR is recommended in all patients with HCM. The evaluation of LGE and the identification of edema help to define both arrhythmic risk and progression to AHF. Indeed, the extension of LGE is associated with a higher mortality for refractory HF [130]. In a histological study of explanted hearts with end-stage HCM, fibrosis involved more than one-third of the LV, particularly the LV apex and the mid-wall [131].

#### 3.2.3. Treatment of HCM

The management of LVOT obstruction is based on drug therapy with beta blockers, calcium channel blockers, disopyramide, and a cardiac myosin ATPase inhibitor (mavacamten) or, if refractory, on invasive surgical or percutaneous septal reduction therapies. Regarding end-stage HCM resulting in severe HF, there are no strong therapeutic recommendations. Current guidelines recommend the same management for HF regardless of the etiology [1]. However, these patients may experience numerous adverse effects of drugs: vasodilators can cause hypotension, while diuretics and negative inotropic drugs used against LVOT obstruction could be poorly tolerated. Furthermore, the current guidelines confirm the indication of primary prevention ICD implantation in patients with a 5-year risk of SCD ≥6% (Class IIa Level B) and with a 5-year risk of SCD ≥4% and <6% (Class IIb Level B). It also advises the consideration of primary prevention ICD implantation in the presence of LGE ≥ 15% or with LVEF <50%, regardless of the risk score (Class IIb Level B) [75].

#### 3.2.4. MCS and HT in HCM

HCM patients who progress to AHF are a difficult population to treat with LVAD therapy, since the small LV cavity and severely impaired diastolic filling pressure may cause inflow cannula obstruction or a risk of ventricular and atrial suction events and lead to low pump flows, VA, and pump thrombosis more frequently. However, a recent analysis evaluated outcomes of RCM and HCM after LVAD, demonstrating that in selected patients with non-obstructive HCM and larger LV cavities, overall survival was similar to DCM patients, with a 1-year survival of 50% on pumps [132]. Since the progression of the disease is rapid from the onset of symptoms to the development of AHF, early inclusion on an HT list should be considered, especially in young patients and patients with a family history, an EF <50%, a severe restrictive diastolic pattern, and a high distribution of LGE [125].

#### 3.2.5. Prognosis of HCM

Patients with end-stage HCM have a very poor prognosis with greater mortality (11% per year vs. 1% per year) compared with the overall HCM population [125]. However, as recently reported, data on survival after HT are reassuring, with 1-, 5-, and 10-year survival rates after HF of 89%, 79%, and 66%, respectively [133].

### 3.3. Restrictive Cardiomyopathy

RCMs are a rare and heterogeneous group of diseases characterized by restrictive left and/or right ventricle pathophysiology in the presence of normal or reduced diastolic and systolic volumes (of one or both ventricles) and normal ventricular wall thickness [75]. The spectrum of RCM includes idiopathic or genetic forms, endomyocardial disorders such as endomyocardial fibrosis and Loffler endocarditis, and myocardial extracellular matrix disorders such as hyperoxaluria and radiation exposition. In addition, myocardial diseases, often in the context of LV hypertrophy, such as amyloidosis, sarcoidosis, or hemochromatosis, can progress to occasional restrictive physiology [75]. Around 30% of patients with RCM have mutations in sarcomeric, cytoskeletal, nuclear envelope, filamin, titin, or desmin genes, which have autosomal dominant patterns of transmission and incomplete penetrance [134]. Rapezzi et al. proposed a classification for RCM according to myocardial histology, genetic basis, and the transient or permanent nature of restriction. They only identify apolipoprotein A (APOA), variant amyloid transthyretin (ATTRv) and AL amyloidosis, genetic RCM, radiation-mediated RCM, tropical and non-tropical endomyocardial fibrosis, and hypereosinophilic syndrome (HES) as “true RCM” [135].

#### 3.3.1. AHF in RCM

RCM is associated with the worst prognosis of all the CMPs. The prognosis of RCM depends on the restrictive physiology, regardless of etiology [136,137]. Indeed, the increased stiffness of the myocardium and the rising filling pressure, without a compensatory increase in volume, may lead to pulmonary and systemic congestion and, in the end, systolic LV dysfunction. The prevalence of HF in RCM patients is high. In a large European registry of CMPs, HF is reported in 83% of RCM patients (present in 41%, 40%, and 1.6% of NYHA class II, III, and IV patients, respectively) [123]. In a cohort of 97 patients with genetic RCM, 81% developed HF, 53% demonstrated NYHA class II symptoms, and 28% demonstrated NYHA class III–IV symptoms [137]. Among children with RCM, more than 75% demonstrate advanced HF and poor prognoses (death or HT within a few years after diagnosis) [136,138]. The clinical features associated with an increased risk of death or transplantation include HF symptoms, a reduced EF, increased left atrial size, and impaired LV diastolic function on echocardiography [138,139,140]. However, available data on AHF in patients with RCM are limited and not fully defined. Only the LeoDOR trial reports a percentage of 2.2% RCM patients in the levosimendan group, but no specific data are available for their outcomes [36]. Considering recent trials on amyloid CMP, tafamidis and, more recently, vutrisiran reduced mortality and HF rehospitalizations. In a multicenter randomized trial of 441 patients with transthyretin amyloid CMP (either variant or wild-type ATTR amyloidosis, of which 76% were wild-type), tafamidis, which binds to transthyretin and prevents tetramer dissociation and amyloidogenesis, was associated with lower all-cause mortality than the placebo (78 of 264 [29.5%] vs. 76 of 177 [42.9%]; HR: 0.70; 95% CI 0.51 to 0.96), as well as a lower rate of CV-related hospitalizations, with a relative risk ratio of 0.68 (0.48 per year vs. 0.70 per year; 95% CI 0.56 to 0.81). Interestingly, heart and liver transplantation and LVAD use were treated as death for the purposes of this analysis. Rehospitalization rates due to CV-related causes were lower with tafamidis but were frequent in both groups (52.3% vs. 60.5%) [141]. Recently, in 665 patients with ATTR-CM (either variant or wild-type ATTR amyloidosis, 89% were wild-type), vutrisiran, a subcutaneously administered RNA interference therapeutic agent that inhibits hepatic synthesis of both wild-type and variant TTR messenger RNA, led to a lower risk of all-cause death and recurrent CV events (defined as hospitalizations for CV causes or urgent visits for HF) compared with the placebo (HR in the overall population, 0.72; 95% CI 0.56 to 0.93; *p* = 0.01). Yet, recurrent hospitalizations for CV-related causes or urgent visits for HF were 34% vs. 41% (HR in the overall population, 0.73; 95% CI 0.61 to 0.88; *p* = 0.001) [142].

#### 3.3.2. Diagnosis of RCM

The diagnosis algorithm should include clinical examinations, an ECG, and cardiac imaging [143]. The most frequent ECG abnormality is AF, particularly in end-stage RCM (>50%) [137]. Echocardiography is essential in advanced stages of RCM demonstrating increased left atrial volume and restrictive LV filling with elevated ventricular filling pressures. CMR is essential for the tissue characterization of the disease, while an endomyocardial biopsy may be indicated in complex cases such as endomyocardial disorders. Right heart catheterization (RHC) may help in differentiating the alternative diagnosis of constrictive pericarditis [75].

#### 3.3.3. Treatment of RCM

The prognosis remains poor despite medical therapy. Due to the restrictive physiology in RCM, standard heart failure treatment requires modification. In patients with pronounced restrictive filling, heart-rate-lowering medications such as beta blockers should be avoided or used with extreme caution due to the risk of hemodynamic deterioration. Similarly, since RCM patients often have low-to-normal blood pressure, drugs acting on the renin–angiotensin–aldosterone system are frequently poorly tolerated due to hypotension. While loop diuretics remain the primary therapy for relieving congestion, careful monitoring is essential to avoid overdiuresis, since even mild hypovolemia can provoke hypotension and hypoperfusion [144].

#### 3.3.4. MCS and HT in RCM

RCM patients are frequently excluded from LVAD implantation due to small cavities and restrictive physiologies that impair pump function [144]. However, in a small cohort of RCM patients treated with LVAD, improved survival was reported, especially among patients with larger LV dimensions [145]. In another cohort of patients, 60% of RCM patients survived at least 1 year after LVAD implantation [132]. HT may be considered for symptomatic RCM patients without pulmonary hypertension or in patients with end-stage HF [144]. During the planning of an HT, an etiological diagnosis is essential in order to identify forms that benefit from specific therapies [75]. In a cohort of 544 RCM patients, the prevalence of HT was 1.4%, with reduced survival after the HT (1-, 5-, and 10-year overall survival after HT of 84%, 66%, and 45%, respectively) [146].

#### 3.3.5. Prognosis of RCM

RCM is associated with the worst prognosis among CMPs. A total of 50% of patients die within two years from diagnosis and the prognosis is worse with increasing NYHA class, irrespective of other characteristics [134].

### 3.4. Non-Dilated Left Ventricular Cardiomyopathy

The 2023 ESC Guidelines for the Management of CMPs have introduced a new CMP subtype called NDLVC. This phenotype is characterized by the presence of non-ischemic LV scarring or fatty replacement in the absence of LV dilatation, with or without global or regional wall motion abnormalities, or isolated global LV hypokinesia without scarring (as assessed by the presence of LGE on CMR) that is unexplained solely by abnormal loading conditions (hypertension, valve disease, or CAD) [75].

#### 3.4.1. AHF in NDLVC

There are no data evaluating the progression to advanced HF in patients with NDLVC. NDLVC patients are a heterogeneous group in which it is necessary to provide thorough characterization and assess clinical outcomes. Indeed, in these patients, who are not dilated and have a normal or less reduced EF, the evolution into AHF is not as concerning as the arrhythmic risk. In a survival analysis of patients with or without dysfunction and with or without LGE, patients with dysfunction (EF < 50%) and LGE showed a lower survival rate than patients without dysfunction and with or without LGE [147]. Castrichini et al. first performed a clinical characterization of patients with NDLVC, demonstrating that septal LGE, together with LV dilatation, age, advanced disease, and frequent and repetitive VAs, was a predictor of major arrhythmic events [148].

#### 3.4.2. Diagnosis of NDLVC

Diagnosis is based on a multimodality imaging approach and includes echocardiography to provide all relevant information on global and regional LV anatomy, function, and hemodynamics, as well as CMR, which is the foremost imaging modality in NDLVC patients as it provides confirmation of the presence of LGE and its localization. Numerous studies have demonstrated the association between LGE and a worse prognosis, emphasizing that a greater extension of the LGE pattern or its distribution at the level of the anterior septum is associated with a worse prognosis regardless of the LVEF [106,149,150]. In line with previous studies, Castrichini et al. demonstrated that the presence of LGE is associated with a high risk of major arrhythmic events and that septal LGE is an independent predictor of outcome, irrespective of the underlying genetic substrate [148]. A recent observational, longitudinal cohort study of 42 patients meeting the criteria for NDLVC demonstrated a heterogeneous etiology. Over one-third of cases had an underlying genetic etiology, nearly one-fifth had an inflammatory or neuromuscular condition, and the remaining cases lacked an identified underlying etiology after a comprehensive diagnostic work-up. A genetic cause was more frequent in patients with LGE at CMR compared with patients with isolated LV systolic dysfunction without fibrosis. Myocarditis-like onset was a common clinical presentation, with nearly one-third of cases uncovering a pathogenic (P)/likely pathogenic (LP) variant in DSP, confirming the emerging notion that myocarditis can uncover a genetic CMP. Finally, while most patients with genetic NDLVC fulfilled diagnostic criteria for arrhythmogenic left ventricular cardiomyopathy (ALVC) according to the Padua criteria, the diagnosis of ALVC was rarely observed in patients with an inflammatory or neuromuscular etiology [151]. Furthermore, the authors of the DERIVATE study, selecting patients with NDLVC criteria, demonstrated that patients with “hypokinetic” NDLVC (without LGE) had significantly lower rates of major adverse arrhythmic cardiac events (MAACE) than those with non-ischemic DCM (*p* = 0.001). However, patients with “fibrotic” NDLVC (with LGE) had similar rates of all-cause mortality (*p* = 0.48) and MAACE (*p* = 0.616) compared with non-ischemic DCM patients [152]. The ESC CMP guidelines’ core message is that phenotype must guide specific disease definitions. These data support the concept that NDLVC might be considered an umbrella term encompassing patients with various etiologies and differing prognoses. The ESC guidelines emphasize that this phenotype is a crucial first step in the diagnostic pathway, not a diagnosis itself, and its identification should trigger an investigation into the underlying etiology [75].

#### 3.4.3. Treatment, MCS, and HT in NDLVC

Validated criteria for the stratification of arrhythmic risk are lacking. However, as for DCM patients, clinical and family history, the extent and localization of LGE, and genetic mutations should be evaluated to consider primary prevention ICD implantation [75]. The clinical management of HF should be based on HF guidelines [1]. HT is the treatment of choice in patients with end-stage HF, whereas LVADs are valid alternatives in patients ineligible for HT or with AHF.

#### 3.4.4. Prognosis of NDLVC

As reported previously, factors associated with poor outcomes are septal LGE, LV dilatation, age, advanced disease, and frequent and repetitive VAs [148]. The evolution into AHF in NDLVC patients is currently unknown.

### 3.5. Arrhythmogenic Right Ventricular Cardiomyopathy

ARVC is a heart muscle disease structurally characterized by progressive myocardial atrophy with fibro-fatty replacement of RV myocardium, causing RV dilatation and/or dysfunction and predisposing to fatal VAs [75]. Over time, the focus of ARVC has moved from a severe RV disease with malignant VAs to a broader concept that includes biventricular or even left-dominant disease [153]. ARVC has a prevalence of 0.078% and 60% of patients inherit gene mutations (mainly in desmosomal genes such as DSP, PKP2, DSG2, DSC2, and JUP) with an autosomal dominant pattern. Patients with multiple mutations have a worse prognosis [75,154].

#### 3.5.1. AHF in ARVC

Three stages of the disease can be described:

(1) First stage: asymptomatic, but still at risk of SCD;
(2) Electrical phase: characterized by symptomatic monomorphic ventricular VT;
(3) Advanced phase: characterized by advanced HF. In this phase, VT and ventricular fibrillation due to fibrosis and reactive inflammation are possible and LV involvement is frequent. Notably, LV involvement in the final stage of the disease is an old concept, as emerging data suggest that it can be found in the early stages of the disease [155]. Only 3.9% of ARVC patients manifest advanced HF, while specific data regarding AHF are scarce and often derived from studies not exclusively focused on this condition [123].

#### 3.5.2. Diagnosis of ARVC

The diagnostic criteria used for recognizing ARVC patients were published by Marcus et al. in 2010 and are still used [156]. The Padua criteria, instead, have offered an integration to include LV involvement [153]. The diagnostic work-up includes ECG (more frequently T-wave inversion in V1–V3, VT, and ventricular ectopic beats with typical left-bundle branch morphology), Holter monitoring, cardiac imaging, genetic testing, and, rarely, endomyocardial biopsy [75]. Echocardiography is essential for recognizing RV dilatation, akinetic–dyskinetic wall motion abnormalities, and myocardial aneurysms and biventricular dysfunction that characterize the evolution to AHF. However, CMR is considered the first-line test for the assessment of the RV’s functional and structural abnormalities [156]. Moreover, subepicardial LGE is typical of ARVC, particularly in cases of DSP variants [156,157].

#### 3.5.3. Treatment, MCS, and HT in ARVC

In ARVC patients who are developing AHF, the recommendations should not deviate from AHF guidelines [1]. ICD implantation is recommended in patients with a history of cardiac arrest or sustained VT and should be considered in patients with high-risk features (arrhythmic syncope, non-sustained VT, RVEF < 40%, LVEF < 45%, or sustained monomorphic VT) [75]. Only a small portion of patients undergo HT, while LVAD could be used as a bridge to HT. However, the involvement of the RV contraindicates the exclusive use of left-side MCS [158].

#### 3.5.4. Prognosis of ARVC

A young age is the only independent predictor of worse outcomes. The main indication for HT is HF and the survival at 5 years is 91% [158].

## 4. Conclusions

AHF is a complex and life-threatening syndrome requiring urgent hospitalization and medical intervention. However, AHF is an extremely heterogeneous umbrella term. Indeed, the rapid onset of symptoms and signs of HF can be due to several causes and can present with different clinical scenarios, thus significantly influencing clinical management and prognosis (Figure 1). For example, the de novo presentation of AHF with pulmonary edema in a patient with a hypertensive emergency or tachi-bradyarrhythmia can often be easily managed and can potentially lead to a better prognosis compared to a patient with CS. Yet, even in the latter context, ACS as a leading cause can lead to a potentially better prognosis compared with a patient with end-stage/advanced HF, as the potential treatment options can solve the root cause behind CS. Within the AHF scenario, patients with specific CMPs are also included; however, CMPs are often under-represented compared to ischemic etiology and are mixed with other etiologies in the so-called “non-ischemic HF” group in many studies on the management of and therapies for chronic or acute HF. Therefore, future studies should be more detailed in exploring the etiology, clinical presentation, treatment, and outcomes of patients presenting with AHF, and specific data on the characteristics of AHF presentation, treatment, and outcomes of individual CMPs are warranted.

## Figures and Tables

**Table 3:** Main recent randomized chronic heart failure trials with rates of ADHF.

	**Patients (*n*)**	**Year of Publication**	**Population Characteristics**	**Randomized Arms**	**Primary Endpoint**	**Rates of ADHF and Main Results**
SENIORS trial [70]	2128	2005	Patients aged >70 years and with a history of HF (hospital admission for HF within the previous year or known EF < 35%)	To receive nebivolol or placebo	A composite of all-cause mortality or CV hospital admission	31.1% of patients in the nebivolol group vs. 35.3% in the placebo group (HR 0.86, 95% CI 0.74–0.99; *p* = 0.039)
PARADIGM-HF trial [67]	8442	2014	Patients who had HF with a reduced EF of 40% or less	To receive either angiotensin receptor–neprilysin inhibitor (at a dose of 200 mg twice daily) or enalapril (at a dose of 10 mg twice daily)	A composite of death from CV causes or hospitalization for HF	(1) 21.8% in the angiotensin receptor–neprilysin inhibitor group and 26.5% in the enalapril group (HR, 0.80; 95% CI, 0.73 to 0.87, *p* < 0.001) (2) Angiotensin receptor–neprilysin inhibitor reduced the risk of hospitalization for HF by 21% (*p* < 0.001) and decreased the symptoms and physical limitations of HF (*p* = 0.001)
COMMANDER HF trial [71]	5022	2018	Patients with worsening chronic HF, a left ventricular EF of 40% or less, and underlying CAD	To receive rivaroxaban at a dose of 2.5 mg twice daily or placebo in addition to standard care after treatment for an episode of worsening HF	A composite of death from any cause, MI, or stroke	(1) 25.0% of patients in the rivaroxaban group and 26.2% of patients in the placebo group (HR, 0.94; 95% CI, 0.84 to 1.05; *p* = 0.27) (2) The composite outcome of CV death or rehospitalization for HF occurred in 37.2% in the rivaroxaban group and in 36.9% of the placebo group (HR, 0.99; 95% CI, 0.91 to 1.09). (3) 27.5% of patients in rivaroxaban group vs. 27.5% in the placebo group (HR, 95% CI, 0.98 (0.89–1.09) had a rehospitalization for worsening HF
MITRA-FR trial [72]	152	2018	Patients with chronic HF with reduced left ventricular EF (15–40%) and severe secondary mitral valve regurgitation	To undergo percutaneous mitral valve repair in addition to receiving medical therapy (intervention group) or to receive medical therapy alone (control group)	Composite of death from any cause or unplanned hospitalization for HF at 12 months	(1) 54.6% in the intervention group and 51.3% in the control group (odds ratio, 1.16; 95% CI, 0.73 to 1.84; *p* = 0.53) (2) The rate of unplanned hospitalization for HF was 48.7% in the intervention group and 47.4% in the control group (HR, 1.13; 95% CI, 0.81 to 1.56)
COAPT trial [73]	614	2018	Patients with HF with reduced EF (20–50%) and moderate-to-severe or severe secondary mitral regurgitation who remained symptomatic despite the use of maximal doses of guideline-directed medical therapy	To transcatheter mitral valve repair plus medical therapy (device group) or medical therapy alone (control group)	All hospitalizations for HF within 24 months of follow-up	35.8% per patient-year in the device group vs. 67.9% per patient-year in the control group (HR, 0.53; 95% CI, 0.40 to 0.70; *p* < 0.001)
DAPA-HF trial [62]	4744	2019	Patients with NYHA class II, III, or IV HF and an EF of 40% or less	To receive either dapagliflozin (at a dose of 10 mg once daily) or placebo, in addition to recommended therapy	A composite of worsening HF (hospitalization or an urgent visit resulting in intravenous therapy for HF) or CV death over a median of 18.2 months	(1) 16.3% of patients in the dapagliflozin group and 21.2% of patients in the placebo group (HR, 0.74; 95% CI, 0.65 to 0.85; *p* < 0.001) (2) A first worsening HF event occurred in 10.0% of patients in the dapagliflozin group and in 13.7% of patients in the placebo group (HR, 0.70; 95% CI, 0.59 to 0.83)
EMPEROR-Reduced trial [63]	3730	2020	Patients with class II to IV HF with an EF of ≤40%	To empagliflozin (10 mg once daily) or placebo in addition to recommended treatments for HF	The composite of CV death or hospitalization for HF during a median follow-up of 16 months	(1) 415 empagliflozin patients vs. 519 placebo patients; (HR, 0.76; 95% CI, 0.67–0.87; *p* < 0.0001) (2) Less HF hospitalizations that required intensive care (HR, 0.67; 95% CI, 0.50–0.90; *p* = 0.008) and that required a vasopressor or positive inotropic drug or mechanical or surgical intervention in the empagliflozin group (HR, 0.64; 95% CI, 0.47–0.87; *p* = 0.005)
SOLOIST-WHF trial [32]	1222	2021	Patients with type 2 diabetes mellitus who were recently hospitalized for worsening HF	To receive sotagliflozin or placebo	The total number of deaths from CV causes and hospitalizations and urgent visits for HF within a median of 9 months	The rate (the number of events per 100 patient-years) of primary endpoint events was lower in the sotagliflozin group (51.0 in the sotagliflozin group vs. 76.3 in the placebo group (HR: 0.67; 95% CI: 0.52 to 0.85; *p* < 0.001))
IRONMAN trial [64]	1137	2022	Patients with HF (EF ≤ 45%) and transferrin saturation less than 20% or serum ferritin less than 100 μg/L	To intravenous ferric derisomaltose or usual care	Recurrent hospital admissions for HF and CV death within a median follow-up of 2.7 years	22.4% of patients in the ferric derisomaltose group and 27.5% of patients in the usual care group (RR: 0.82 [95% CI 0.66 to 1.02]; *p* = 0.070)
DIAMOND trial [66]	1642	2022	Patients with HF and reduced EF and current or a history of RAASi-related hyperkalemia	To receive Patiromer or placebo	Mean change in serum potassium from baseline over a median follow-up of 27 weeks	(1) +0.03 mmol/l in the patiromer group and +0.13 mmol/l in the placebo group [−0.10 mmol/l (95% CI −0.13, 0.07); *p* < 0.001] (2) Total HF hospitalizations were 17 out of 439 in the patiromer group vs. 20 out of 439 in the placebo group (HR, 95% CI 0.79 (0.36, 1.71) *p* = 0.544)
TRANSFORM-HF trial [65]	2859	2023	Patients hospitalized with HF (regardless of EF)	To receive torsemide or furosemide	All-cause mortality over a median follow-up of 17.4 months	(1) 26.1% of patients in the torsemide group and 26.2% of patients in the furosemide group (HR, 1.02 [95% CI, 0.89–1.18]) (2) All-cause mortality or all-cause hospitalization occurred in 47.3% of patients in the torsemide group and in 49.3% in the furosemide group (HR, 0.92 [95% CI, 0.83–1.02]) (3) 940 total hospitalizations among 536 participants in the torsemide group and 987 total hospitalizations among 577 participants in the furosemide group (RR, 0.94 [95% CI, 0.84–1.07])
TRILUMINATE trial [74]	350	2023	Patients with severe tricuspid regurgitation, symptomatic, in NYHA class II, III, or IV, a pulmonary artery systolic pressure of less than 70 mm Hg, and in stable (≥30 days) guideline-directed medical therapy for HF	To receive either TEER or medical therapy (control)	Hierarchical composite of death from any cause or tricuspid valve surgery, hospitalization for HF, and an improvement in quality of life as measured with KCCQ at the 1-year follow-up	11,348 wins for the TEER group, 7643 wins for the control group, and 11,634 ties between the groups (win ratio: 1.48 (95% CI, 1.06 to 2.13; *p* = 0.02). The annualized rate of hospitalization for HF was 0.21 events per patient-year in the TEER group vs. 0.17 events per patient-year in the control group
RESHAPE HF2 trial [68]	505	2024	Patients with HF and moderate-to-severe functional mitral regurgitation	To either transcatheter mitral valve repair and guideline-recommended medical therapy (device group) or medical therapy alone (control group)	(1) The rate of the composite of first or recurrent hospitalizations for HF or CV death during 24 months (2) The rate of first or recurrent hospitalizations for HF during 24 months (3) The change from baseline to 12 months in the score on the KCCQ-OS	(1) 37 events per 100 patient-years in the device group and 58.9 events per 100 patient-years in the control group (RR, 0.64; 95% CI, 0.48 to 0.85; *p* = 0.002) (2) 26.9 events per 100 patient-years in the device group and 46.6 events per 100 patient-years in the control group (RR, 0.59; 95% CI, 0.42 to 0.82; *p* = 0.002) (3) The KCCQ-OS score increased by a mean (±SD) of 21.6 ± 26.9 points in the device group and 8.0 ± 24.5 points in the control group (mean difference, 10.9 points; 95% CI, 6.8 to 15.0; *p* < 0.001)
MATTERHORN trial [69]	210	2024	Patients with HF and secondary mitral regurgitation who have symptoms despite guideline-directed medical therapy	To undergo either transcatheter edge-to-edge repair (intervention group) or surgical mitral valve repair or replacement (surgery group)	A composite of death, hospitalization for HF, mitral valve reintervention, implantation of an assist device, or stroke within 1 year after the procedure	(1) 16.7% of patients in the intervention group vs. 22.5% in the surgery group (estimated mean difference, −6 percentage points; 95% CI, −17 to 6; *p* < 0.001 for noninferiority) (2) Rehospitalization because of congestive HF: 1.0% in the interventional group vs. 3.5% in the surgery group (difference (95% CI) −3 (−9 to 3)

ADHF: acutely decompensated heart failure; CAD: coronary artery disease; CI: confidence interval; CV: cardiovascular; EF: ejection fraction; HF: heart failure; HR: hazard ratio; MI: myocardial infarction; KCCQ-OS: Kansas City Cardiomyopathy Questionnaire–Overall Summary; NYHA: New York Heart Association functional class; RAASi: renin–angiotensin–aldosterone system inhibitor; RR: rate ratio; TEER: tricuspid transcatheter edge-to-edge repair.

## Data Availability

Not applicable.

## Abbreviations

ACS	acute coronary syndrome
ADHF	acutely decompensated heart failure
AF	atrial fibrillation
AHF	acute heart failure
ALVC	arrhythmogenic left ventricular cardiomyopathy
AKI	acute kidney injury
APOA	apolipoprotein A (APOA)
ARVC	arrhythmogenic right ventricular cardiomyopathy
ATTRv	variant amyloid transthyretin
CAD	coronary artery disease
CCS	chronic coronary syndrome
CHD	congenital heart disease
CMP	Cardiomyopathy
CMR	cardiac magnetic resonance
CO	cardiac output
COPD	chronic obstructive pulmonary disease
CS	cardiogenic shock
CVD	cardiovascular disease
DCM	dilated cardiomyopathy
DSP	desmoplakin
ESC	European Society of Cardiology
FLCN	filamin C
GDMT	guideline-directed medical therapy
eGFR	Glomerular Filtration Rate
HCM	hypertrophic cardiomyopathy
HES	hypereosinophilic syndrome
HFpEF	heart failure with preserved ejection fraction
HFrEF	Heart Failure with Reduced Ejection Fraction
HF	heart failure
HT	heart transplant
ICD	Implantable Cardioverter Defibrillator
ICU	intensive care unit
LGE	late gadolinium enhancement
LMNA	lamin A/C
LP	likely pathogenic
LV	left ventricular
LVAD	Left Ventricular Assist Device
LVEF	left ventricle ejection fraction
MAACE	Major adverse arrhythmic cardiac events
MCS	Mechanical circulatory support
MI	myocardial infarction
M-TEER	mitral transcatheter edge-to-edge repair
NDLVC	non-dilated left ventricular cardiomyopathy
NYHA	New York Heart Association Functional Classification
P	pathogenic
PE	pulmonary embolism
PLN	phospholamban
RCM	restrictive cardiomyopathy
RHC	Right heart catheterization
RMB	RNA binding motif protein
RV	right ventricular
SBP	systolic blood pressure
SCD	sudden cardiac death
VA	ventricular arrhythmia
VT	ventricular tachycardia
